# Single Nucleotide Polymorphism Charting of *P. patens* Reveals Accumulation of Somatic Mutations During *in vitro* Culture on the Scale of Natural Variation by Selfing

**DOI:** 10.3389/fpls.2020.00813

**Published:** 2020-07-07

**Authors:** Fabian B. Haas, Noe Fernandez-Pozo, Rabea Meyberg, Pierre-François Perroud, Marco Göttig, Nora Stingl, Denis Saint-Marcoux, Jane A. Langdale, Stefan A. Rensing

**Affiliations:** ^1^Plant Cell Biology, Department of Biology, University of Marburg, Marburg, Germany; ^2^Department of Plant Sciences, University of Oxford, Oxford, United Kingdom; ^3^Université de Lyon, UJM-Saint-Etienne, CNRS, Laboratoire BVpam - FRE 3727, Saint-Étienne, France; ^4^BIOSS Centre for Biological Signalling Studies, University of Freiburg, Freiburg, Germany; ^5^SYNMIKRO Center for Synthetic Microbiology, University of Marburg, Marburg, Germany

**Keywords:** SNP, RNA-seq, *Physcomitrella patens*, *Physcomitrium*, ecotype, Gransden, Reute, RFLP

## Abstract

**Introduction:** *Physcomitrium patens* (Hedw.) Mitten (previously known as *Physcomitrella patens*) was collected by H.L.K. Whitehouse in Gransden Wood (Huntingdonshire, United Kingdom) in 1962 and distributed across the globe starting in 1974. Hence, the Gransden accession has been cultured *in vitro* in laboratories for half a century. Today, there are more than 13 different pedigrees derived from the original accession. Additionally, accessions from other sites worldwide were collected during the last decades.

**Methods and Results:** In this study, 250 high throughput RNA sequencing (RNA-seq) samples and 25 gDNA samples were used to detect single nucleotide polymorphisms (SNPs). Analyses were performed using five different *P. patens* accessions and 13 different Gransden pedigrees. SNPs were overlaid with metadata and known phenotypic variations. Unique SNPs defining Gransden pedigrees and accessions were identified and experimentally confirmed. They can be successfully employed for PCR-based identification.

**Conclusion:** We show independent mutations in different Gransden laboratory pedigrees, demonstrating that somatic mutations occur and accumulate during *in vitro* culture. The frequency of such mutations is similar to those observed in naturally occurring populations. We present evidence that vegetative propagation leads to accumulation of deleterious mutations, and that sexual reproduction purges those. Unique SNP sets for five different *P. patens* accessions were isolated and can be used to determine individual accessions as well as Gransden pedigrees. Based on that, laboratory methods to easily determine *P. patens* accessions and Gransden pedigrees are presented.

## Introduction

Single Nucleotide Polymorphisms (SNPs) represent a major source of natural variation within any given species. In the plant kingdom, they are studied both in ecological and evolutionary context in order to understand population structure (Leaché and Oaks, 2017). They are also employed to study the genetic basis of variable natural traits such as resistance to flooding (Vashisht et al., 2011), or for the identification of genetic diversity in cultivars and admixed wild types through association mapping (Niu et al., 2019). SNP analysis is now successfully integrated in plant breeding for example in palm tree selection (Xia et al., 2019). For the moss model *Physcomitrium patens* (Hedw.) Mitten (previously known as *Physcomitrella patens*) (Beike et al., 2014; Medina et al., 2019; Rensing et al., 2020) whole genome SNP sets between the reference genome accession, Gransden (Gd) (Rensing et al., 2008; Lang et al., 2018), and the accessions Villersexel (Vx) (Kamisugi et al., 2008), Reute (Re) (Hiss et al., 2017) and Kaskaskia (Ka) (Perroud et al., 2011) have been reported (Hiss et al., 2017). Specifically, the genetic difference between Gd and Vx has been used to generate the first sequence-anchored genetic linkage map (Kamisugi et al., 2008) and recently the *P. patens* chromosome level genome assembly (Lang et al., 2018). Analysis of SNP segregation is a powerful tool that can be employed to analyze intra and inter accession fertility (Perroud et al., 2011, 2019; Meyberg et al., 2020), gene specific segregation patterns, and loci affected in segregants with specific traits. For example, the analysis of Gd and Vx segregants has been used to identify the *ANR* locus affected in mutants impaired in ABA hormone signaling (Stevenson et al., 2016), as well as loci involved in three-dimensional morphogenesis [*nog*1, (Moody et al., 2018)] and a novel microtubule depolymerizing-end-tracking protein [*CLoG*1, (Ding et al., 2018)]. Most recently, SNPs between Gd and Re were associated with the loss of fertility in the Gd background (Meyberg et al., 2020). However, there is no comparative study on a broad set of accessions, or within the different *P. patens* Gransden laboratory strains (Gd pedigrees).

Model organisms cultivated in the laboratory are usually considered to be genetically uniform due to their common origin. The original *P. patens* Gransden plant was collected by H.L.K. Whitehouse in Gransden Wood (Huntingdonshire, United Kingdom) in 1962. Engel cultured Whitehouse’s sample (Engel, 1968) and derived the ancestor of all current *P. patens* Gransden strains from a single spore. In 1974 progeny of *P. patens* Gransden started to be distributed across the globe (Ashton and Cove, 1977; Cove, 2005). Since then, *P. patens* became an important model organism *inter alia* to study cell biology, evolutionary developmental biology and the water to land transition of plant life (Rensing, 2018; de Vries and Rensing, 2020). During its decades of *in vitro* cultivation, *P. patens* Gransden was predominantly propagated vegetatively (Ashton and Raju, 2000). While many labs vegetatively propagate the plants, others regularly let the plants go through the life cycle (sexual reproduction through selfing) and establish fresh cultures based on single spores. However, for most of the pedigrees the frequency and number of sexual reproduction events the plants went through is unknown. Phenotypic differences are documented between laboratory strains, for example Gransden strains have shown different levels of loss of fertility (Meyberg et al., 2020). This recently led to the introduction of the Reute accession for the study of sexual reproduction (Hiss et al., 2017). Mutations underlying such differences as well as potential silent mutations can occur during sexual as well as vegetative propagation in the lab. Such laboratory divergences haven been reported in both prokaryote (Smits, 2017) and eukaryote laboratory models, for example in *Chlamydomonas reinhardtii* (Flowers et al., 2015). Mutation and selection underlie the forces of evolution. However, under laboratory conditions natural selection usually is absent. Over time, somatic mutations can thus accumulate in laboratory strains that would not occur in natural populations. Indeed, repetitive vegetative propagation of *P. patens* in the laboratory loosens the selection pressure on genes required for sexual reproduction, apparently leading to deterioration of the latter (Ashton and Raju, 2000; Perroud et al., 2011; Hiss et al., 2017; Meyberg et al., 2020). It should be noted that *P. patens* is predominantly selfing in the (dominant) haploid stage, developing completely homozygous diploid sporophytes. Hence, spores result that are genetically identical to the parent even though they are the product of meiosis.

Previous *P. patens* SNP studies analyzed genomic DNA samples of different *P. patens* accessions (Hiss et al., 2017; Lang et al., 2018). However, *P. patens* gDNA samples are rare. Nevertheless, the recent publication of RNA-seq datasets (Demko et al., 2014; Frank and Scanlon, 2015; Stevenson et al., 2016; Szövényi et al., 2017; Perroud et al., 2018; Fernandez-Pozo et al., 2019) provides a source of information that can be used to detect SNPs. Due to the high number of RNA-seq samples analyzed, efficient pipeline processing is essential. A framework of a modular RNA-seq pipeline was previously published (Perroud et al., 2018). While adding to and modifying this pipeline, a powerful solution for the here presented SNP analysis was created. Due to the current lack of genomic DNA we analyzed whether the SNP analysis of RNA-seq samples leads to comparable results. Based on the called SNPs we determined the rate and nature of somatic mutations among the accessions and pedigrees.

To identify and track genetic variation in the laboratory, restriction fragment length polymorphisms (RFLP) can be employed. This technique is based on SNPs modifying restriction enzyme recognition sites, which are covered by polymerase chain reaction (PCR) amplicons to test for genetic variation in specific DNA regions (Botstein et al., 1980).

Here, we identified SNPs using recently published RNA-seq data as well as unpublished RNA-seq and gDNA-seq data for a range of *P. patens* accessions and Gd pedigrees, i.e., laboratory strains with a documented ancestry. We used the resulting data to separate accessions as well as pedigrees via SNP analysis, extracted unique SNP sets for all accessions and Gd pedigrees, and developed RFLP analyses that are useful in maintaining accession and Gd pedigree identification.

## Materials and Methods

### Sequence Sources

This study used data of five different *P. patens* accessions: 171 Gransden (Gd), 20 Kaskaskia (Ka), 32 Reute (Re), 27 Villersexel (Vx), and 25 Wisconsin (Wi) samples. The dataset contains 206 previously published RNA-seq samples as well as 44 novel RNA-seq samples. In addition, 25 novel gDNA samples of *P. patens* accession Wisconsin (Wi) were analyzed. These 275 samples were used for SNP detection. In addition, the Wi gDNA samples were used to study variation in a naturally occurring population. All samples used in the present study are available at the NCBI SRA database and are detailed in Supplementary Table S1.

### Plant Material, Nucleic Acid Extraction and Sequencing

*Physcomitrella patens* accession Villersexel was collected in 2003 by M. Lueth in Haute-Saone (France) on dry mud at a fish pond east of Villersexel, at the Villers la Ville junction (voucher 4296). The accession Kaskaskia was also collected in 2003 in Illinois (United States) on a periodically flooded drainage channel at a corn field by D. Vitt and M. Sargent. The voucher information for both accessions has previously been published (von Stackelberg et al., 2006; Beike et al., 2014). Accession Reute has also been collected by M. Lueth/M. von Stackelberg in 2006 close to Freiburg, Germany on an agriculturally used field. The exact location has previously been published (Hiss et al., 2017).

#### Reute Early Sporophyte 1 (ES1)

*Physcomitrella patens* accession Reute_2015 (Re_2015) (Hiss et al., 2017) was cultivated on 9 cm petri dishes on solid Knop’s medium enclosed with parafilm under long day conditions (70 μmol m^∗^−2 s^∗^−1 white light, 16 h light, 8 h dark, 22°C) as described in Hiss et al. (2017). Re was regularly reproduced sexually once per year since 2011. Re_2015 is the culture derived from the sexual reproduction (selfing) performed in 2015. Gametangia induction was performed by transfer to short day conditions (see Hiss et al., 2017 for culture details). Sporophytes were harvested 6–9 days after watering and immediately put into 50 μl RNA-later (Qiagen, Hilden, Germany). RNA was extracted using 20 ES1 sporophytes (according to Hiss et al., 2017) using the RNeasy micro kit (Qiagen, Hilden, Germany), following the manufacturers’ protocol. RNA concentration and quality were analyzed with the Agilent RNA 6000 Nano Kit on a Bioanalyzer 2100 (Agilent Technologies). Library preparation and subsequent sequencing was performed by the Max-Planck-Genome-Centre Cologne (mpgc.mpipz.mpg.de). A single library was prepared using the IVT-based low input RNA-seq protocol followed by sequencing with Illumina HiSeq 3000 (150 nt, single ended).

#### Kaskaskia RNA-seq

*Physcomitrella patens* accession Kaskaskia was isolated from seven days entrained protonemal culture under long day conditions (70 μmol m^∗^−2 s^∗^−1 white light, 16 h light, 8 h dark, 22°C), if not stated otherwise (Supplementary Table S2). Tissue was flash frozen in liquid nitrogen and the subsequent RNA extractions were performed as described in (Perroud et al., 2018). The library preparation and subsequent sequencing was processed using the TruSeq RNA kit (Illumina) according to the manufacturer’s instructions. The libraries were sequenced with Illumina HiSeq (100 nt, paired-end).

#### Villersexel Laser Capture of Sexual Reproduction Stages

*Physcomitrella patens* Villersexel (Vx) plants were routinely grown under sterile conditions on ammonium supplemented medium under 20 μmol m^∗^−2 s^∗^−1 of continuous light at 24°C. Protonemata were obtained from ground tissue and cultivated on cellophane disks on the previous medium. After 2 weeks, small patches of protonemata were transferred to low nitrate medium and grown for about 2 months under 20 μmol m^∗^−2 s^∗^−1 of a 16:8 light:dark cycle at 24°C. Well-developed gametophores were then transferred to 16°C under the same light regime for 3 weeks to induce sexual organ differentiation. Fertilization was synchronized in all cultures by flooding growing pots with sterile deionised water for 30 h; flooded gametophores were transferred to 24°C under continuous light. 48 h after flooding, gametophore tips were examined under a hand dissection microscope for the presence of fertilized archegonia. Non-fertilized cultures were treated as previously except for flooding.

Fertilized and unfertilized archegonia were hand dissected and collected in 100% acetone. Tissue fixation was ensured by infiltrating archegonia under low pressure for 2 min followed by a 48 h incubation in 100% acetone. Acetone was then exchanged with HistoClear by incubating fixed tissues in 50% acetone/50% HistoClear for 2 h then 100% HistoClear for 2 h under continuous shaking. Tissues were embedded in wax using an automated Tissue Tek VIP 5 Vacuum Infiltration (Sakura) machine with the following sequence: 3 baths in HistoClear for 1, 1 and 2 h then 4 baths in wax for 1, 1, 2 and 2 h. Thick sections of 10 μm were prepared from the embedded tissues and deposited on Nuclease-free 1.0 polyethylene naphthalate (PEN) membrane slides (Carl Zeiss Microscopy, #415190-9081-000) in drops of 1 X ProtectRNA^TM^ RNase Inhibitor (SIGMA #R7397), air dried and stored at room temperature until further use. After wax removal in HistoClear and 100% ethanol baths, zygote/early embryos, egg cell and archegonium tissues were laser dissected from the sections using a PALM MicroBeam unit (Carl Zeiss) at a 40x magnification following the procedure described in Saint-Marcoux et al. (2015). About 200 sections were captured per sample and 3 biological replicates were prepared for each tissue.

RNA was extracted using the PicoPure RNA extraction kit from Life Technologies (#KIT0204) and amplified into cDNAs using the Ovation RNA-Seq System v2 kit from NuGEN (#7102-32) as in Saint-Marcoux et al. (2015). cDNA quantity was determined using a NanoDrop ND-1000 spectrophotometer. cDNA quality was analyzed on a 2100 BioAnalyzer (Agilent Technologies) using RNA nano chips (5067-1511, Agilent Technologies) following recommendations in the NuGEN kit.

1μg of cDNA was paired-end sequenced on an Illumina HiSeq 2000 platform at the Beijing Genomics Institute in China. At least 2 × 10 million 100 nt reads were obtained per sample. Samples containing “orphans” in the sample name contain reads where the mate did not pass the quality filter.

#### Wisconsin gDNA

Mature (brown) spore capsules of *Physcomitrium patens* were collected in September 2017 in Wisconsin, United States (original specimen in AUGIE herbarium) by Rafael Medina (Augustana College Illinois). The surface sterilization procedure was performed at a laminar flow bench with freshly prepared 1% sodium hypochlorite and autoclaved tap water for rinsing. Five Single spore capsule were sterilized separately. After the last rinsing step the water was kept in the tube and the spore capsule was squeezed by sterile forceps so that the spores were released into the water. This spore suspension was transferred (using a micro pipette and autoclaved filter-tips) to solidified (0.9% [w/v] agar) Knop’s medium containing 1% glucose in 9 cm Petri dishes sealed using 3M Micropore tape or Parafilm. After 3–5 days, when spore germination starts, five single sporelings were isolated from each capsule batch and separately transferred to fresh plates. After eight weeks under long-day conditions juvenile gametophores (above agar) were harvested and immediately frozen in liquid nitrogen. Genomic DNA was isolated from frozen plant material as previously described (Lang et al., 2018). Library-preparation and sequencing was performed at the Max-Planck-Genome-Centre Cologne (mpgc.mpipz.mpg.de); 25 TPase-based DNA libraries were sequenced in 1 × 150 bp single reads on Illumina HiSeq 3000 Analyzers.

Wisconsin experiment 2 was contaminated by prokaryotic sequences. The read contamination removal was done as described in Lang et al. (2018) and Nguyen et al. (2019). The leftover reads were used for further analysis.

### Read Analysis

**FIGURE 1:**
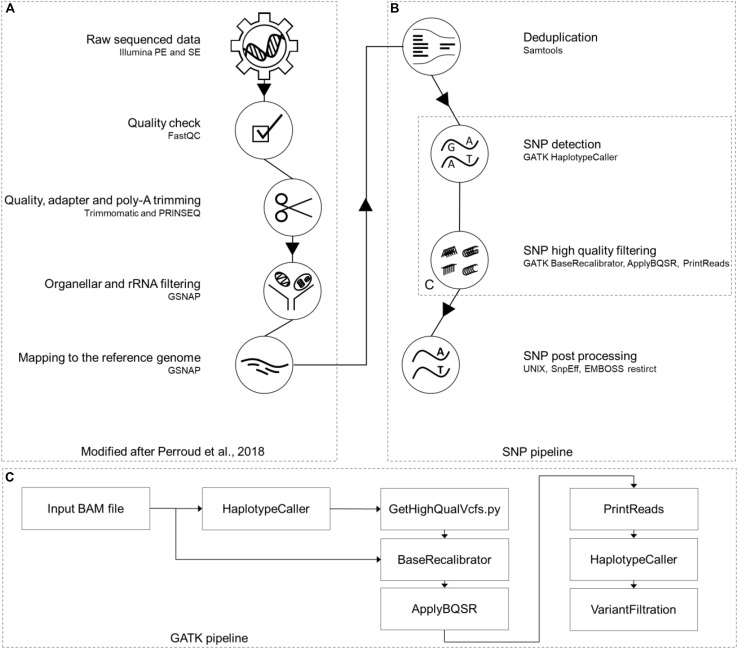
RNA-seq SNP calling pipeline. Part **(A)** of this pipeline was previously published (Perroud et al., 2018). The additional SNP calling branch **(B)** on the right side starts with removing read duplications, using Samtools package “markdup” and continues with the GATK toolbox for SNP calling **(C)**. The last steps of this pipeline are post processing steps like SnpEff and EMBOSS restrict together with UNIX shell scripts. This figure has been modified based on a figure published in The Plant Journal (Perroud et al., 2018; https://onlinelibrary.wiley.com/doi/full/10.1111/tpj.13940).

For easier manageability of the data, all original sample names were converted to a new nomenclature. Separator is always an underscore; the first two characters identify the accession (Gransden [Gd], Reute [Re], Kaskaskia [Ka], Villersexel [Vx] and Wisconsin [Wi]), the next one the origin/pedigree of the sample (e.g., MR-WT11), followed by the experiment defined by roman numbers (e.g., XX). Sample replicates (1-5), library type (SE or PE) and experiment type (mutant [MUT] or wild type [WTY]) are the last parts (Supplementary script “rename and extraction”, Supplementary File 1). An example sample name is Gd_MR-WT11_XX_1_PE_WTY. Each RNA-seq sample went through a modified pipeline, build on top of the RNA-seq pipeline previously described (Perroud et al., 2018). The pipeline was modified by updating all software versions, enabling single-end (SE) read processing and adding SNP calling and post processing parts (Figure 1).

#### Read Quality

For read quality filtering and adapter removal, Trimmomatic (Bolger et al., 2014) version 0.39, was used. Adapter trimming of appropriate adapters (SE.fna or PE.fna; standard sequences included in the Trimmomatic package) was performed with a seed mismatch of 2, a palindrome clip threshold of 30, and a simple clip threshold of 10 for the paired-end reads (PE.fna:2:30:10). Base pairs with a quality score less than three were removed from the start (LEADING:3) and end (TRAILING:3) of the reads. Reads were further filtered using a sliding window of four base pairs with a minimum average quality score of 15 (SLIDINGWINDOW:4:15), removal of the first 10 base pairs (HEADCROP:10), and kept reads of 30 base pairs or more (MINLEN:30).

Poly-A clipping was performed by Prinseq-lite (Schmieder and Edwards, 2011) version 0.20.4. A minimum length of five poly-A/T nucleotides at the 5′- or 3′-end were required to remove the poly-A/T tails (TRIM_TAIL_LEFT 5; TRIM_TAIL_RIGHT 5). Only reads longer than 30 nt were kept (min_len 30).

#### Reference Genome Mapping

All filtered RNA-seq samples were mapped to the *P. patens* reference genome V3 (Lang et al., 2018) by GMAP-GSNAP (Wu and Nacu, 2010) version 2018-7-04. SAM and BAM file processing was performed by samtools (Li et al., 2009) version 1.9. Only uniquely mapped reads were used for further analysis.

#### Removing Duplicate Reads

De-duplication based on the unique mapped BAM files was done using samtools package markdup with the remove duplicate reads option (r).

### Variant Detection

The SNP calling pipeline (Figures 1B,C) uses GATK version 4.0.9.0 (McKenna et al., 2010). The workflow was setup according to the classic GATK best practices workflow for RNA-seq^1,2^ by modification of the approach published earlier (Hiss et al., 2017).

#### SNP Calling

GATK HaplotypeCaller was performed in default mode. To account for *P. patens* being haploid, the option “ploidy 1” was used.

The python script GetHighQualVcfs.py (Wang et al., 2012) was used for quality score recalibration. The option for haploid genomes (ploidy 1) was chosen. In addition, the alternative nucleotide quality (ALTQ) needed to be higher than 90% (percentile 90) and the genotype quality (GQ) value had to be greater than 90 (GQ 90).

The GATK tools BaseRecalibrator, ApplyBQSR and PrintReads were used in default mode.

##### Ploidy test

To test the samples’ ploidy, GATK HaplotypeCaller was performed in default mode for diploid genomes (ploidy 2).

The python script GetHighQualVcfs.py was used for quality score recalibration. The option for diploid genomes (ploidy 2) was chosen. In addition, the alternative nucleotide quality needed to be higher than 90% (percentile 90) and the genotype quality (GQ) value had to be greater than 90 (GQ 90).

The results of both ploidy runs (1n and 2n) were compared. The results were interpreted taking into account the knowledge of previously haploid tested samples (Supplementary Table S10; cf. Results). We observed that the differences in the defined genotypes (GATK output 0/0, 0/1, 1/1, and 1/2) correspond to the differences in the number of called SNPs. Therefore, we chose the number of called SNPs to compare the two ploidy runs.

#### Filtering Wisconsin gDNA SNPs

Single nucleotide polymorphisms called from the Wisconsin accession gDNA were filtered by using only reads uniquely mapping to the *P. patens* v3.3 gene annotation (to make the data comparable to the RNA-seq data). Bedtools intersect (Quinlan and Hall, 2010) version 2.29.0 was used, with the option (u) to write the original entry only once if multiple overlaps are found, to extract all gene models intersecting SNPs (Supplementary script “rename_and_extraction”).

#### Post SNP Calling Filter

The JGI gene atlas samples contain spike-in RNAs, which should not harbor SNPs. Hence, based on SNPs detected in these reads, filters were adjusted so that none of the RNA-seq spike-in base changes (sequencing errors) pass it. Filter values were allelic depths for the reference and alternative alleles (AD), mapped read depth (DP) as well as their fold change (FC) plus a minimum of three samples per SNP.

The above described values were adjusted through three consecutive filter steps. (i) The first filter was the read coverage filter with a minimum read depth of nine reads and a minimum of seven reads supporting the SNP. FC of AD and DP has to be greater than 0.77 (Supplementary script “SNP_filtering”). (ii) The second filter step removes all SNPs not present in at least three samples. This filter ensures the use of SNPs found by all technical triplicates of an experiment. (iii) While the third filter removed all indel positions.

The GO bias analyses were conducted as described previously (Widiez et al., 2014) to contrast gene sets affected by SNPs vs. the background of all genes. Visualization of the GO terms was implemented using word clouds generated by https://www.wortwolken.com. Word size is proportional to the –log10 (*q*-value), and over−represented GO terms were colored dark green if –log10 (*q*-value) ≤ 4 and light green if –log10 (*q*-value) > 4.

Plots were done by using R version 3.6.2 and ggplot2 version 3.2.1. Upset plot for the SNP intersection was performed with the R package UpSetR (Conway et al., 2017). All regression lines and confidence intervals were calculated by the R package ggplot2, method “lm” and the R package ggpubr version 0.2.5 to calculate R^1^ ^,2^.

#### SNP Normalization

Several plots (Supplementary Figures S3–S6) were generated to check for potential normalization methods. The number of read covered base pairs (coverage), the number of reads per sample (reads), and the number of genes, respectively their accumulated length (genes) were taken into account.

##### Coverage method

The dependency of called SNPs based on the number of read covered based pairs was determined with the following method.

To find all read covered base pairs, the mapping output (BAM format) was analyzed by samtools package depth. All sequence positions, including unused reference sequence positions, were printed (aa). The output was filtered for depth ≥ 9 (similar to the SNP DP value). The number of filtered SNPs were divided by the number of read covered base pairs. To compare the values directly with the results found in the division was done vice versa to derive the format “one SNP per X bp”.

To plot the values, the number of SNPs were corrected by the maximum number of read covered base pairs (Supplementary Figure S4).

##### Reads method

To detect the relation between the number of filtered SNPs and the number of sequenced reads, the values were plotted using the R packages described in section “Post SNP Calling Filter.”

##### Genes methods

To answer the question whether SNPs accumulated at specific chromosomes and to observe the relation between the number of genes or their length with the number of detected SNPs, gene information extracted from the *P. patens* v3.3 annotation GFF file (Lang et al., 2018) was used. Both, the number of genes and the gene length, were summarized per chromosome. The extracted gene values were divided by the number of filtered SNPs to derive relation in the gene number and gene length plots, respectively. To test for significance Fisher’s exact test was performed. The number of base pairs w/o SNPs for each of the 27 individual chromosomes (and for all unassigned, merged scaffolds) was compared. All *p*-values were corrected using the R method p.adjust using the method (Benjamini and Hochberg, 1995).

#### Extracting Exclusive SNPs

In the context of SNPs found only in a specific accession or Gd pedigree, the terms unique and exclusive are used synonymously. Exclusive SNPs were extracted for each accession and for each Gransden pedigree, using bash/awk scripts (Supplementary scripts “rename_and_extraction”, “SNP_clustering” and “SNP_filtering”). First, all SNPs found in all GATK VCF files were grouped into a single file. Subsequently, the groups were inspected for SNPs exclusive for a specific accession or Gd pedigree (Supplementary script “SNP_filtering”). For further accession analysis, the SNPs were sorted by the number of supporting samples. SNPs supported by > 90% of the samples of one accession, and not found in others, were defined as exclusive. The read coverage filter was not applied for the accession exclusive SNP selection. For the Gd pedigrees, the Gd exclusive SNPs were ranked by the number of supporting samples. The SNP with most sample support received the highest rank, the five SNPs with the most sample support were chosen and defined as exclusive.

#### Accession Clustering

Detected nucleotide variation was clustered by two different methods. The first method was an artificial FASTA alignment (Supplementary File 4). This method clusters only SNPs, no InDels. Only SNPs that passed all filter steps were used. Each SNP is a single column in the alignment. If the sample contains a SNP at a specific position, the SNP nucleotide was added to the FASTA sequence of the sample, otherwise the reference nucleotide was used.

The second method was chosen to cluster SNPs and InDels. Instead of nucleotides, numbers were chosen to represent a SNP, InDel or the reference. A matrix was created by substitution of reference and variant nucleotides: reference 0; SNP 1; indel 2. This converted numbers were added to the matrix similar to the nucleotides in the above described FASTA file. Each row is a single sample and each column a unique SNP/indel position (Supplementary script “SNP_clustering”).

The artificial FASTA alignment was imported to SplitsTree (Huson and Bryant, 2005) version 4.14.8. A network was calculated using default parameters. The tree was generated by the NJ option and stored in NEXUS format. FigTree (Bouckaert et al., 2014) version 1.4.4 was used to draw a circular tree based on the SplitsTree NEXUS file.

The SNP/indels 0-1-2 matrix was loaded into R version 3.6.2 using the function dist with the method euclidean. To get a three dimensional PCA plot, the results were transferred to the R package rgl version 0.100.30.

#### SNP Effects

Synonymous and non-synonymous SNPs for each sample were detected by SnpEff (Cingolani et al., 2012) version 4.3T in default mode. SnpEff used a database created of the *P. patens* genome annotation v3.3 to locate SNP positions at gene regions. Only SNPs that passed all three filter steps (minimum nine reads have to cover the SNP position and minimum seven reads have to support the SNP, at least three samples have to support the SNP, indels are removed) were used.

Synonymous and non-synonymous SNPs were extracted from the SnpEff CSV file output and all involved genes were extracted from the SnpEff gene.TXT file. Functional analyses were done via GO-bias analysis, described in chapter “Post SNP Calling Filter.”

### Identification of Restriction Sites Overlapping With SNPs

EMBOSS restrict^3^ was used to detect SNPs in putative restriction endonuclease recognition regions. The enzyme database, containing all necessary information about the recognition sites, was loaded with the tool EMBOSS rebaseextract^4^. The rebase restriction endonucleases databases, withrefm.907 and proto.907, were downloaded at ftp://ftp.neb.com/pub/rebase. EMBOSS restrict was performed with a minimum length of the restriction enzyme recognition site of five base pairs (sitelen 5) and all enzyme at the database were used (enzymes all).

### SNP Verification via PCR and RFLP (Restriction Fragment Length Polymorphism)

Exclusive SNPs for each *P. patens* accession overlapping with a restriction enzyme recognition site were selected as described above. SNPs affecting six or eight nt long recognition sites were chosen. Additionally, enzyme requirements for easy usability and frequency of cuts in ± 2 kbp around the SNP were analyzed to ensure an interpretable gel band pattern. Primers were designed to result in an amplicon of 700-1,400 bp and similar annealing temperatures (∼ 59°C, Supplementary Tables S7, S8).

#### Plant Material and gDNA Extraction

To analyze SNPs located within restriction enzyme sites (comparison of accessions) and SNPs without restriction enzyme site (comparison of Gd pedigrees) the *P. patens* accessions and Gd pedigrees Gransden DE Marburg 2015 (Gd_DE_MR), Gransden Japan (Gd_JP, Gd_JP_Okazaki and Gd_JP_St.Louis), Gransden Grenoble (Gd_CH), Reute 2015 (Re), Kaskaskia (Ka) and Villersexel (Vx) were cultivated as described above. Genomic DNA for PCR amplification was isolated, using a fast protocol using one to two gametophores as published in (Cove et al., 2009).

#### PCR Analysis and Sequencing

Polymerase chain reaction was carried out with OneTaq polymerase (NEB) following the manufacturers’ protocol. Annealing was carried out between 55°C and at 59°C and elongation time was adjusted to the longest fragment chosen (95 s). For primer sequences see Supplementary Tables S7, S8. 5 μl PCR product, 2.5 μl of the forward primer (10 μM) and 2.5 μl water were Sanger sequenced (Macrogen, Germany) (Supplementary Table S9 and Supplementary File 6). PCR products and all subsequent fragment analyses were visualized via gel electrophoresis (0.7% agarose, Roth, Germany) using peqGREEN (VWR, Germany) as dye. The 1 kbp size standard was purchased from NEB.

#### Restriction Analysis

For each tested SNP, 15 μl PCR product of all accessions were used as input for the enzymatic digestion. Restriction was carried out for the SNPs Re_c3_17747483_A-T, Vx_c3_2712099_A-G and Ka_c01_25061888_C-A using 2U of the corresponding enzyme (Supplementary Table S7, NEB) for 3 h at 25°C for *Swa*I and at 37°C for *Nde*I and *Xba*I. Fragments resulting from the restriction were visualized via gel electrophoresis as described before (PCR analysis and sequencing).

### Natural Population Diversity

To determine variation within a naturally occurring *P. patens* population, the accession Wisconsin gDNA SNP results were used. Because of bacterial contamination, sample Wi_2 was excluded from this study. The experiment was designed with four capsules and five spores each. Each spore represents one sample. The number of exclusive SNPs for each sample (spore) within a spore capsule were detected as well as the number of exclusive SNPs for each spore capsule. The results were compared with the results of exclusive SNPs found in laboratory accessions and pedigrees of Gransden, Gd_DE 2011, 2012 and 2015, and Reute 2007, 2012 and 2015. To highlight the results Venn diagrams were created by venny^5^.

All samples described above were used to generate an artificial FASTA alignment (for methods see section “Extracting exclusive SNPs”) which was analyzed by Splitstree. Here, only gDNA SNPs which intersected with the *P. patens* v3.3 annotation file were kept. The branch lengths were adjusted by coverage normalization (see section “Coverage method”).

## Results

### Read Analysis and SNP Discovery

The analysis was conducted with a total of 4.7 billion RNA-seq reads (Supplementary Table S3). 68% of all reads are from Gransden, Reute reads account for 18%, Kaskaskia for 12% and Villersexel for 2% (Supplementary Table S4). After pre-processing and mapping to the reference genome (Figure 1A) 81% of all reads remained (Supplementary Table S3). De-duplication (to account for potential PCR bias) further reduced the amount of reads by 20%, leaving 3.0 billion reads as input for the GATK SNP pipeline (Figures 1B,C). The unfiltered Wisconsin gDNA samples amounted to 1.0 billion reads. Processing, mapping to the reference and deduplication discarded more than half of the raw reads; 473 million reads were used for the SNP pipeline (Supplementary Table S3).

Funariaceae are known for naturally occurring polyploidization (Rensing et al., 2013; Beike et al., 2014), this has also been demonstrated during *P. patens* mutant generation using protoplasts (Schween et al., 2005). We performed a ploidy test using GATK with *n* = 1 vs. *n* = 2 and generally detect a lower number of SNPs when assuming haploidy (*n* = 1), on average 65.4% of *n* = 2. The percentage range of samples confirmed to be haploid (36.2 – 92.2%) approximately coincides with the percentage range of all samples (30.7 – 92.9%) (Supplementary Table S10 and Supplementary File 7). Moreover, manual inspection of the VCF files for the Wi gDNA SNP calls showed very minor differences, that are smaller than those of the RNA-seq data of confirmed haploid plants. Taken together, we do not find evidence for polyploid plants among the samples used.

For the Wisconsin gDNA samples 2,473,107 SNPs were called by the GATK pipeline (Figure 1C). After intersecting the gDNA SNPs with the gene coordinates of the *P. patens* v3.3 annotation, 140,832 SNPs were kept that represent the transcriptome, to be comparable to the RNA-seq SNPs. Merging the Wi v3.3 SNPs with the results of the RNA-seq accessions ended up in a total number of 1,233,585 transcribed gene space SNPs. Gd has the lowest number of SNPs relative to the reference assembly. This fits the expectation, since the reference genome was derived from a Gd pedigree. The accessions Wi and Ka have the highest number of SNPs per sample (Supplementary Figure S1). The highest SNP reduction can be observed after the (i) read coverage filter, which was, together with the (ii) sample support filter, adjusted using spike-ins (see section “Materials and Methods” for details). (i) Read coverage and (ii) sample support filter, together with the (iii) indels removal, were reducing the SNP set by 88% (146,816 SNPs shared by five accessions, Supplementary File 5). A comparison of SNP intersection between SNPs called in this study and SNPs previously published (Lang et al., 2018) demonstrates a large overlap of 89% of the previously called Vx SNPs (as compared to those that were detected in this study) and minor overlaps for Re (26%) and Ka (28%) (Supplementary Table S5).

### SNP Comparison Between Accessions

Most SNPs can be observed in the intergenic regions (up- and downstream of the gene bodies according to the v3.3 annotation). The SNP distribution for all accessions is around 40:60 (gene regions/intergenic regions). The accessions Wi and Vx have almost no SNPs flanking the two base pairs next to the splice site (splice site region).

**FIGURE 2:**
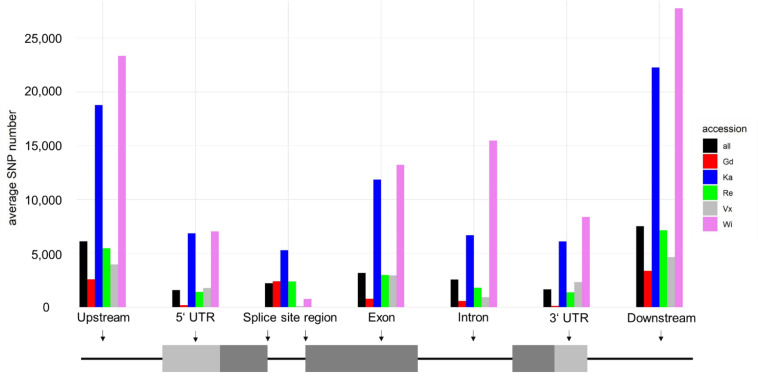
Average snpEff output for each accession. Shown are average numbers of SNPs affecting specific regions, highlighted in a schematic gene structure shown below the corresponding grouped columns. Most SNPs are up- and downstream of genes (intergenic). SNPs at splice site regions are intron SNPs, located on the first and last two intron base pairs.

Most of the SNPs shown in Figure 2 are accumulated in non-coding regions. Exonic SNPs can be synonymous, not affecting the coding sequence, or non-synonymous, leading to a change in the amino acid sequence of the protein encoded by the gene (for average number of SNPs per sample see Table 1 and for total numbers of SNPs see Supplementary Table S11). The two accessions from North America, being geographically most far away from the reference sample, are the ones with the most changes affecting the coding sequence. Individual SNP effects in the exclusive accession SNPs list can be found in Supplementary File 3.

**TABLE 1:** Average number of SNPs affecting gene coding sequences per sample.

	Gd	Re	Vx	Ka	Wi	all
Start changes^a^	3	8	8	18	29	8
Stop changes^b^	6	23	22	97	237	41
Sequence changes^c^	774	2,978	2,942	11,794	13,090	3,168
Synonymous	411	1,232	1,272	4,698	4,737	1,300
Non-synonymous	363	1,746	1,670	7,096	8,353	1,868

*^*a*^Start changes include start codon gains and losses. ^*b*^Stop changes include gains and losses of stop codons. ^*c*^Sequence changes include non-synonymous changes affecting the encoded amino acids, synonymous sequence changes, and insertions or deletions that do not change the sequence frame.*

**FIGURE 3:**
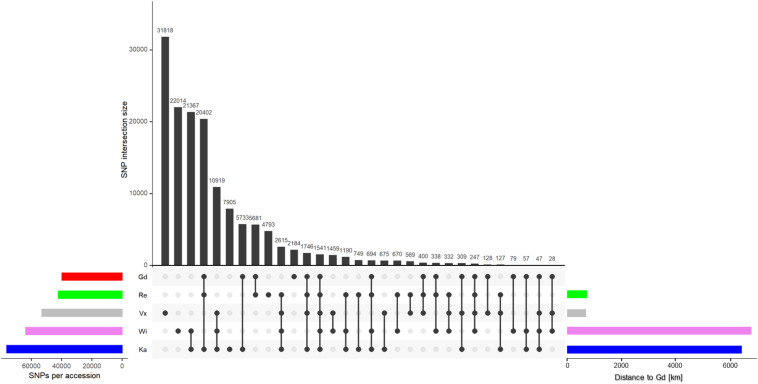
SNP intersection of the five accessions. The horizontal colored bars on the left show the total number of SNPs per accession after applying all three filter steps. The bars to the right show the geographic distance to the reference Gd. The colors represent the five accessions throughout the text. The vertical black bars show the number of intersecting SNPs, marked by the dots below.

Less than 12% of all SNPs called by the GATK pipeline passed all three filter steps: Gd has 39,614 and Re has 42,094 SNPs left, Vx has 52,960 SNPs and Wi has 63,597 SNPs. The highest number of SNPs are found in Ka with 76,076 SNPs (Supplementary Figure S1 and Figure 3, left horizontal bars). The number of SNPs coincides with the geographical distance to the reference Gransden (Figure 3, horizontal bars; Supplementary Figure S2). After applying four different normalization methods (see section “Materials and Methods” for details), Gransden and Reute exhibit always the lowest SNP rate (Supplementary Table S6), mirroring previous results (Beike et al., 2014; Lang et al., 2018). The approximate linear relationship between number of reads and called SNPs (Supplementary Figure S3) led to the normalization by read number. The coefficient of determination (R^2^) is found to be 0.6 – 0.93 (Supplementary Figure S3). To compensate for unequal distribution of reads we also normalized by the fraction of the sequence space that carries enough read support to allow SNP calling (see section “Coverage method,” Supplementary Figure S4). By applying the SNPs to read covered base pairs, instead of the raw read number, the R^2^ values increased. Wi, Ka and Vx reach almost 1, Re and Ge 0.77 and 0.85. Based on the coverage normalization (Supplementary Figure S5), Gd has 1 SNP per 4,666 bp, Reute has 1 SNP per 1,912 bp followed by Ka (1 SNP per 630 bp), Wi (1 SNP per 206 bp) and Vx (1 SNP per 143 bp). The gene normalization methods (Supplementary Figure S6) indicate that chromosome 19 and chromosome 26 exhibit significantly (*q* ≤ 0.05) more SNPs than the other chromosomes.

The SNP intersection shows 1,541 SNPs are shared by all accessions (Figure 3). There are accession specific SNPs (exclusive SNPs) as well. Most exclusive SNPs are present in Vx (31,818), followed by Wi (22,014), Ka (7,905), Re (4,793) and Gd (2,184) (Figure 3, vertical black bars). Gd is sharing 94% of its SNPs with other accessions, Ka and Re share > 87%, Wi shares 65% and Vx 40% SNPs with all other accessions.

Applying a filter to extract exclusive SNPs supported by ≥ 90% of the samples, Wi and Ka have most exclusive SNPs/InDels, Wi has 4,007 unique SNPs, Ka 3,393. 890 SNPs are only present in the Re accession while in the Vx accession 21 exclusive SNPs were found (Supplementary File 3).

100 kbp SNP hotspot regions were detected to survey the *P. patens* accessions (Supplementary Figure S7 and Supplementary File 2). On Chr26, starting at 300,000 bp, a hotspot region is present in all accessions. All accessions but Gd share one region on Chr19. Gd, Re and Ka share 100 kbp hotspot regions on Chr03 and one on Chr06. Ka, Wi, and Vx share regions on Chr04, 07 and 13 (Supplementary Figure S7 and Supplementary File 2). Biased GO terms of the described regions are shown in Supplementary Figure S8. Most 100 kbp SNP hotspot regions are overlapping with the SNP hotspots found by (Lang et al. (2018); Supplementary File 2, Table B). However, there are also a few hotspot regions only found in the present study.

**FIGURE 4:**
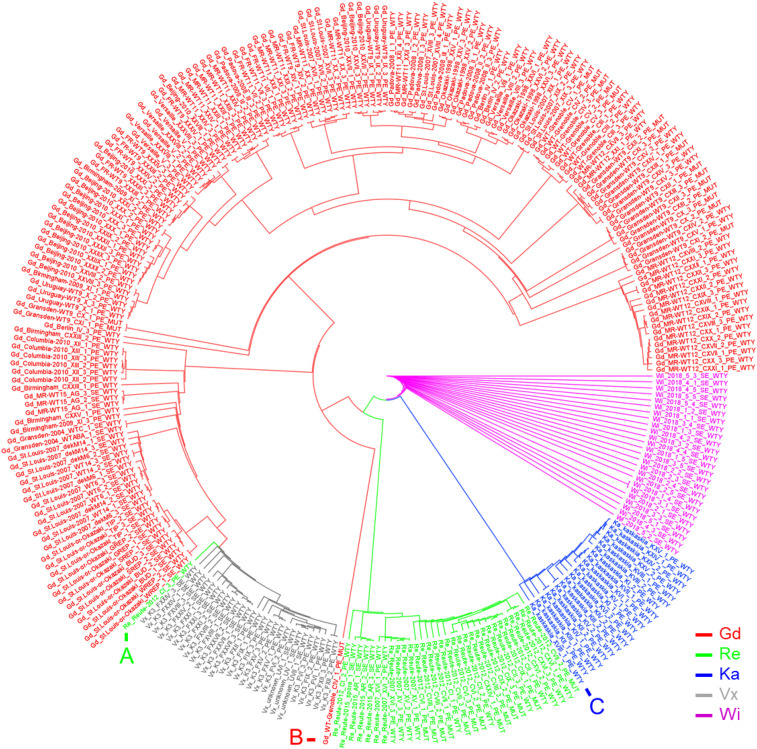
Circularized SplitsTree network based on an artificial FASTA SNP alignment file. The Neighbor-Joining tree of five *P. patens* accessions is shown. All libraries cluster within their accession and applied treatment, except for the marked libraries: **(A)** Sample Re_REUTE-2012_CI_3 has 100 x lower read coverage than the other Re samples. It clusters next to the low read coverage Vx samples. **(B)** Sample Gd_WT-Grenoble_CIV_1 is a Gd outgroup. **(C)** Ka sample which was falsely annotated as Gd at the NCBI SRA (XVIII_1_PE_WTY), determined by exclusive SNP analysis (Supplementary File 3, Sheet Ka_exclusive_SNPs).

Using an artificial FASTA alignment of all SNPs, we performed a clustering analysis (Figure 4). Samples of the accessions Gd, Re, Ka, Vx and Wi are clustering with each other, respectively, indicating that our approach is able to detect the respective genetic background. The three European accessions form a clade to which Ka and Wi are sister. One Re sample, belonging to the experiment CI_3 (NCBI BioProject PRJNA411193), does not cluster with the other Reute samples (Figure 4A). The number of reads in this sample is 100 x lower than in the other samples of experiment CI, potentially causing biased SNP calling and hence incorrect clustering. The Gd sample CIV_1 (Figure 4B) possesses an outlier position with regard to the other European samples. The sample of the NCBI BioProject PRJNA411163 is annotated as Gransden accession. However, it could be shown by clustering (Figure 4C) and exclusive SNP analysis that the sample belongs to the accession Kaskaskia. Principal component analysis (PCA) of SNPs as well as InDels recapitulates the SNP clustering results (Supplementary Figure S9). The samples from Szövényi et al. (2017) went into the SNP calling pipeline as a blind test. The sample origin was originally marked as unknown. Both clustering methods assigned the samples to Vx, with corresponds to the origin confirmed by the authors.

### SNP Comparison of Gransden Pedigrees

**FIGURE 5:**
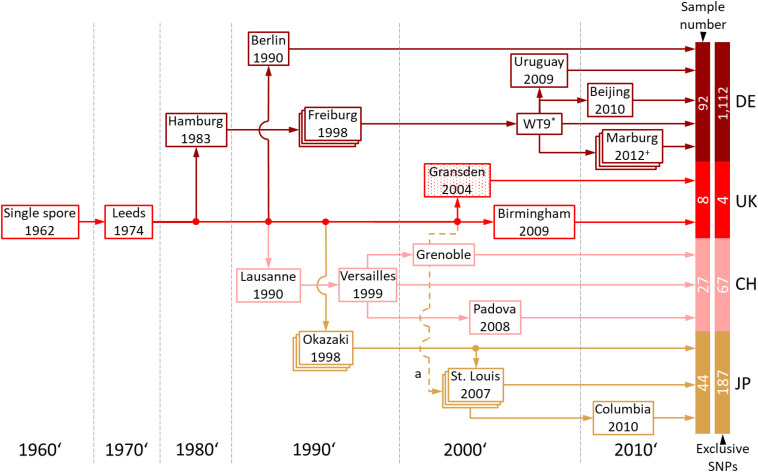
Gransden pedigree. The pedigree diagram shows Gransden strains of 13 different labs used in the present study. The Gransden accession was arranged in four different pedigrees, Germany (DE), United Kingdom (UK), Switzerland (CH) and Japan (JP). The United Kingdom pedigree was sent to St. Louis, United States in 2004 and used to sequence the reference genome (a). However, this strain was not used or broadly distributed afterwards. The plants analyzed in St. Louis are derived from the Japan pedigree (2007). Pedigrees shown in stacked boxes went through yearly selfing. *+* Since 2011 yearly selfing except 2013. * Since 1999 Gransden Freiburg went through nine generations leading to WT9. The numbers of samples and exclusive SNPs for each of the four pedigrees are shown to the right (also shown in Supplementary Figure S11).

Gransden is more widely used in laboratories than any of the other *P. patens* accessions. Based on information retrieved from the laboratories involved, the Gransden accession was classified into four pedigrees, Germany (DE), United Kingdom (UK), Switzerland (CH) and Japan (JP) (Figure 5). The original Gransden accession from the United Kingdom made it first to Hamburg, Germany (founding the DE pedigree), before it was sent to Lausanne, Switzerland (CH) and Okazaki, Japan (JP). The Lausanne strain was sent to Versailles, France and further distributed to Padova, Italy and Grenoble, France. In 1998, Gransden DE arrived in Freiburg, Germany. In Freiburg the Gd plants went through sexual reproduction (selfing) once per year. Starting 1999 the Freiburg pedigree went through nine rounds of selfing leading to WT9. The offspring were labeled by consecutive numbers or the year of sexual propagation. Gransden Freiburg (WT9) was sent to Uruguay, Beijing (China) and Marburg, Germany. Gransden Marburg started in 2011 and went through selfing each year except 2013. The Gd United Kingdom 2004 sample was sent to St. Louis, United States (Figure 5a) for gDNA isolation and used to sequence the *P. patens* reference genome (Rensing et al., 2008). However, the Gd UK 2004 reference sample was not broadly distributed. In 2007, another Gd sample was sent to St. Louis, USA from Okazaki, Japan. These plants were used for further analysis and also sent to Columbia. It should be noted that most papers that cite the reference genome paper with its Gd 2004 sample are actually using different pedigrees.

Our analyses show that Gransden accumulated different mutations in different laboratories during prolonged *in vitro* culture. To eliminate misleading SNP background noise, the exclusive SNPs for the Gd pedigrees were detected after applying read coverage and sample support filters. The intersection of the four Gd pedigrees (Supplementary Figure S11) shows that Gransden Germany (DE) has 1,112 exclusive SNPs while Gd_CH has 67 exclusive SNPs, Gd_JP 187 and Gd_UK features four (Figure 5). Because there is no SNP supported by at least 90% of all samples of a specific pedigree, the extraction of exclusive SNPs was done by getting the best supported SNPs. SNP ranking by the number of samples that support it was used to select the five most supported SNPs for a given pedigree. The Gd_DE top five SNPs are supported by 76–77 samples, Gd_CH between 12 and 18 samples, Gd_JP by 12 to 29 samples. For Gd_UK three samples support the top five list (Supplementary File 3). A clear clustering based on the FASTA alignment file, as for the accessions, is not possible (Supplementary Figure S10). In some cases, the samples grouped by experiments instead of Gd pedigree, which could be due to the low number of SNPs, and similar genes being expressed, biasing the number of available SNPs for the comparisons. If samples are highly specific for a single tissue (e.g., antheridia bundles or spores), not all genes are covered by the extracted transcripts and consequently SNPs cannot be detected.

Since some of the samples have a documented sexual propagation history (i.e., we know how many years/cycles of sexual reproduction lie between samples) we used the opportunity to determine whether SNPs were generally lost or gained in these samples. We find that for samples that were subject to regular sexual reproduction, SNP numbers generally decreased along the timeline (Supplementary Table S12 and Supplementary Figure S12). The observed mutation rate was found to be similar across the different pedigrees (Supplementary Table S13).

### Experimental Confirmation of Selected SNPs via Sequencing and RFLP Analysis

**FIGURE 6:**
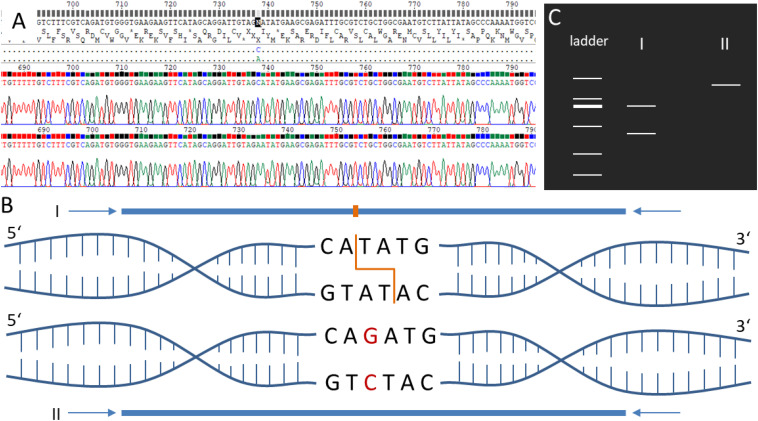
Schematic visualization of the restriction fragment length polymorphism (RFLP) analysis. **(A)** Electropherogram of the sequenced amplicons generated via PCR using forward and reverse primers. **(B)** PCR amplicons of the samples I and II covering the same genomic position in two different *P. patens* accessions. Sample I sequence includes a restriction enzyme site for *Nde*I (orange). Sample II contains a single nucleotide polymorphism (SNP, red) resulting in the loss of the restriction enzyme site. **(C)** If amplicons are digested via the corresponding restriction enzyme Nde*I*, sample I results in two bands when separated via gel electrophoresis, whereas sample II results in one band. See Supplementary Figures S13–S15 for experimental verification of the accession-specific RFLP regions.

For all primer pairs (Supplementary Tables S7, S8) covering SNPs specific for different accessions, PCR amplicons could be generated. Sequencing analysis of the PCR products showed in all tested positions (9/9 positions, Supplementary Tables S7, S8) the presence of the predicted SNP in the corresponding accessions’ and Gd pedigree background (Supplementary Figures S13–S17). To provide an easy and cheap tool to distinguish the different accessions, RFLP analysis (Figure 6) was successfully established for the SNPs Re_c3_17747483_A-T, Vx_c3_2712099_A-G and Ka_c01_25061888_C-G (Supplementary Figures S13–S15). The Re_c3_17747483_A-T amplicon (1,255 nt) was digested with *Nde*I resulting in two fragments (990 nt and 265 nt) for the accessions Gd, Ka and Vx, and absence of digestion in Re (Supplementary Figure S13). For Vx_c3_2712099_A-G, the amplicon of 1,366 nt was digested with *Swa*I leading to two fragments (1,063 nt and 303 nt) in Gd, Ka and Re but not in Vx (Supplementary Figure S14). For Ka_c01_25061888_C-G, the 1,342 nt amplicon was digested with *Xba*I resulting in two fragments (984 nt and 358 nt) in Gd, Re and Vx, but no digestion in Ka (Supplementary Figure S15). Results for SNPs not tested by RFLP (Supplementary Table S8) for two accession primer pairs (Re_c04_21933417 and Vx_c13_4764050) and five Gd pedigree primer pairs (Gd_DE_c02_12750876, Gd_DE_c05_3105395, Gd_DE_c12_2095061, Gd_JP_c20_868 8243, Gd_CH_c23_11248087), show the presence of the predicted accession and Gd pedigree SNPs on the sequence level (Supplementary Figures S16, S17).

### Natural Population Variation and Selection

Samples of pedigrees with known propagation history were chosen to estimate the annual number of mutations per base pair (observed mutation rate). The time period covered is six years for Gd and eight years for Re. The number of SNPs called for all pedigrees generally decreases under regular sexual propagation. The same is true for the estimated mutation rate (Supplementary Table S13). The lowest annual mutation rate with 2E-07 was detected for the Freiburg WT11 (FR_WT11) pedigree, the highest rate for Reute-2012 with 4E-06.

**FIGURE 7:**
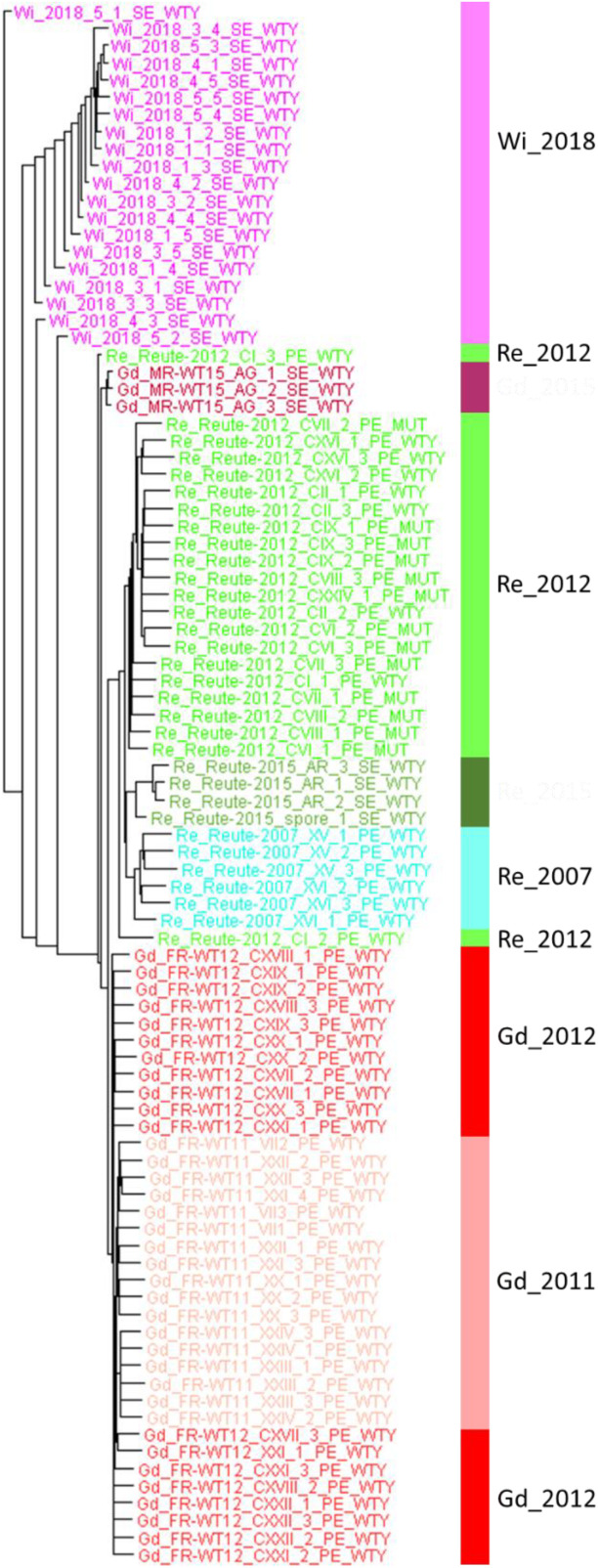
Splitstree tree of Wisconsin natural population and three generations of Gd and Re. The tree is based on part of the artificial SNP FASTA alignment containing Wi samples (without the bacterial contaminated spore capsule experiment 2) and three generations of Re (2007, 2012 and 2015) and Gd (2011, 2012 and 2015). The Splitstree network tree was branch length-corrected by the maximum number of covered base pairs (see coverage normalization in section “Materials and Methods”).

The diversity of genome-wide SNPs found within the Wisconsin natural population single spore isolates is lower compared with three selfed generations (pedigrees) of laboratory accessions. The lower numbers can be observed both on sample/spore and on pedigree/capsule level (Supplementary Figure S18). However, on the level of the artificial FASTA alignment of the gene body SNPs, represented by a Splitstree tree (Figure 7), similar normalized branch lengths for Wi samples and most Re and Gd pedigrees can be observed.

The ploidy test using GATK with *n* = 1 and *n* = 2 resulted in a high rate of congruence for Wi. The *n* = 1 explained 84.3% – 95.6% (average 88.1%) of the SNPs called in the *n* = 2 run (Supplementary File 7). Approximately 18% of the Wi SNPs are heterozygous, a lower number than for any of the other accessions/pedigrees (Supplementary Table S14). Hence, the naturally occurring heterozygosity of the Wi population is lower than that observed in cultured samples. Much of what is detected as heterozygous is probably due to very closely related (identical and near-identical) paralogs that are known to be present in the *P. patens* genome (Rensing et al., 2008). Yet, the low apparent Wi heterozygosity reinforces that *P. patens* is a predominantly selfing species (Perroud et al., 2019; Meyberg et al., 2020; Rensing et al., 2020).

We calculated the rate between non-synonymous nucleotide changes (Ka) and synonymous changes (Ks) per sample and accession (Supplementary Table S11 and Supplementary File 7). Over all samples, the Ka/Ks rates follow a clear linear trend (R^2^_adj_ = 0.98, Supplementary Figure S19), suggesting neutral evolution (no global selective pressure). However, most individual samples deviate from the 99% confidence interval of the linear regression and hence putatively show evidence of negative selection (Ks ≫ Ka), or positive (Darwinian) selection (Ka ≫ Ks). The accession Gd, which represents the genome reference, apparently is under negative selection, all the other four accessions show evidence of positive selection (Supplementary Table S11 and Supplementary File 8). The GO bias of genes affected by non-synonymous changes was calculated and visualized via word clouds (Supplementary Figure S20).

## Discussion

### Read Analysis and SNP Discovery

Here, we analyzed sequence variants in *P. patens* accessions and Gransden pedigrees using mainly sequences from gene expression (RNA-seq) experiments. Therefore, this study is limited to the gene space, lacking information of most of the intergenic regions, where the selection pressure is lower and more changes accumulate (Krasovec et al., 2017). On the other hand, the advantage of using RNA-seq data is the much higher availability of data. Very few genomic data sets, and with low sequencing depth, are currently available for *P. patens* accessions and Gransden pedigrees. However, hundreds of RNA-seq experiments could be used in this study, allowing much higher resolution to detect sequence variants in genes. To ensure the quality of the SNPs found, several filters were applied. Finding a feasible filter for the called SNPs is a major step during the analysis due to risk of over- or underestimation. The presence of RNA spike-ins in some of the samples, which mimic natural eukaryotic mRNAs, gave us the opportunity to distinguish sequencing/mapping errors from actual sequence variants.

Single nucleotide polymorphisms filtering is required to reduce the false-positive rate of SNP detection. Amplification errors during sample preparation and sequencing (Ma et al., 2019) can lead to incorrectly called SNPs as well as software issues while mapping and SNP calling (Ribeiro et al., 2015). We used RNA spike-ins to detect such false-positive SNPs. Spike-ins do not exhibit SNPs. Hence, all called SNPs in spike-in mRNAs represent sequencing or computation errors. The read depth filter was adjusted to remove spike-in SNPs without losing too much sensitivity. GATK output VCF files contain a lot of information about the background data of the SNP, *inter alia*, read coverage at the SNP position. By extracting all spike-in SNPs and evaluating different parameters, the read coverage parameter [DP] and the parameter of how many reads at that position were supporting the SNP [AP], seemed to be the most feasible parameters to filter out spike-in SNPs. The number for DP of nine reads was chosen because only 4/381 spike-in SNPs were left after applying that filter (equaling 1% false positives; at DP = 10 the sensitivity breaks down). Another observation led to the sample support filter. SNP variation of more than 30% between replicate RNA-seq samples could be observed (Supplementary Figure S18,A). Using only SNPs found in at least three samples removed the last four false positive spike-in SNPs and makes the remaining SNPs more reliable. The improvement of filtering can also be observed by comparing the results with previously detected SNPs. The intersection of SNPs called in this study and SNPs found by Lang et al. (2018) shows an increasing number of intersection by applying the three filtering steps (Supplementary Table S5). The SNPs found for Re and Ka maybe have been under-estimated by Lang et al. (2018). The accessions Re and Ka have a 10 x lower number of SNPs compared to the accession Vx (Lang et al., 2018). Here, the number of intersecting SNPs between (Lang et al., 2018) and our results shows an almost 90% intersection of Vx SNPs at the strictest filter step. For Re and Ka, the intersection is less than 30% (Supplementary Table S5). Potentially, the absence of Re and Ka SNPs in the previous study is a result of sub optimally adjusted filter parameters or it could be an effect of low read coverage. Sufficient read depth at library level, large number of read mapping/coverage and high sequencing quality are major foundations for high quality SNP calling results. In some cases, it is possible that some SNPs were not found in one accession or strain because the data available for that position and accession was not enough to detect it in a reliable way. Samples with low read coverage show inconsistency in SNP-to-read correlation (Supplementary Figures S3–S5). A reason for this behavior could be non-linear relation between number of SNPs and number of reads for very high and very low read numbers. Samples with a low number of reads can lead to incoherent SNP calling results due to stochastic coverage fluctuation. The high variability in such low read coverage samples can be observed in Supplementary Figure S5: the data range of Wi and Vx are wider than all the others. The low number of reads available for the Vx laser capture experiment (BioSample PRJNA602303) is probably related to the RNA-seq extraction technique, yielding small amounts of RNA that might be prone to bias before and/or after amplification.

To reduce the SNP per read effect, we normalized the SNPs by the coverage method, resulting in an observable increase of linear relationship (Supplementary Figures S3, S4). The number of SNPs called for each sample became more reliable in terms of comparability and reflect well previous studies and expectation of genetic distance coinciding with geographic distance (Supplementary Figure S2). The RNA-seq based SNP pipeline described here can in future be applied to stringently call SNPs for *P. patens* accessions and pedigrees, or can be adjusted to suit data sets from other model organisms for which a reference genome or transcriptome is available.

### SNP Comparison Between Accessions

When locating the position of the SNPs in the genome, most of them were found in non-coding regions upstream and downstream the gene body (UTRs), as well as in introns and splicing sites within the introns. Many changes were observed in the coding sequences of the five accessions. These changes may lead to alterations in the protein sequence of the final gene product, by changing start or stop codons, or producing frame changes (Table 1).

The total number of filtered raw SNPs per accession (Figure 3) in comparison to the Gd genome reference shows (as expected) the Gd accessions as the one with the smallest number of changes followed by Re, Vx, Wi and Ka. This order agrees with the distance to the Gd geographical location in the Southeast of England (Supplementary Figure S2): Re (Hiss et al., 2017) and Vx (Kamisugi et al., 2008) in close vicinity to each other at the border of France and Southwestern Germany, and Wi and Ka (Perroud et al., 2011) in North America.

Results from Lang et al. (2018), where variance at genomic level was detected using the accessions Re, Vx and Ka, showed a SNP rate of one SNP per 1,783 bp for Re, per 644 bp for Ka and 188 bp for Vx while another study found a SNP rate of one SNP per 207 bp for Vx (Ding et al., 2018). Similar results for the number of base pairs per SNP can be found for the RNA-seq analysis in this study (Re 1 SNP each 1,912 bp, Ka 630 bp and Vx 143 bp) (Supplementary Figure S5). The SNP density based on RNA-seq (this study) and gDNA (Lang et al., 2018) is similar, although more SNPs are expected to be detected based on gDNA due to the presence of intergenic regions that are not under selection. This could be another indication of an underestimated SNP number as discussed above. In any case, our method using RNA-seq data for gene space SNP calling yields appropriate results allowing to estimate differences in accessions by SNPs.

We have chosen two different methods to cluster the SNPs related to each sample. An artificial FASTA alignment with all SNPs as well as a matrix including SNPs and indels. Both methods show similar results (Figure 4 and Supplementary Figure S9). The outlier sample Re_CI_3 has a very small read number, probably yielding misleading results. Sample Gd_CIV_1 also appears as an outlier (Figure 4). However, in the PCA 3D plot, the sample clusters according to expectation (Supplementary Figure S9). Our SNP pipeline had proven its functionality by blind tests as well as by pointing out unexpected metadata errors. The sample Ka_XVIII_1 was re-sequenced to replace a previous Gd experiment in which one of the triplicates failed (Perroud et al., 2018). For this sample, our SNP clustering (Figure 4) shows clear evidence for the accession being Ka, not Gd. Indeed, manual checking exclusive SNPs there is no doubt that it is Ka (Supplementary File 3, Sheet Ka_exclusive_SNPs). Most probably, the plant material was accidentally mislabeled.

The extraction of exclusive SNP sets for each of the five accessions helps to identify unknown *P. patens* sequences. Here we provide a set of SNPs for all examined accessions that will be useful for molecular identification of accessions. The low number of exclusive Vx SNPs are based on the uniqueness of the single Vx samples. Each Vx sample provided a big list of SNPs, but a high number of these SNPs were only available in one or two other Vx samples. A higher read coverage or more standardized mRNA could solve this issue. For low coverage reasons, we were not using the read coverage filter for the detection of exclusive SNPs. High sample support was chosen as an alternative and promoted exclusive SNP selection in a reasonable way, yielding confirmable molecular identification.

Observed approximate linearity between number of called SNPs and reads per sample (Supplementary Figure S3) lead to the read normalization method. When applying the coverage method that takes into account the fraction of the gene space covered by enough reads to allow SNP calling, linearity increased even further (Supplementary Figure S4). While both raw and normalized counts lead to the same conclusions in terms of genetic distance, we suggest the coverage normalization to most accurately describe the data.

### SNP Comparison of Gransden Pedigrees

Gd is the current reference accession for *P. patens*, and was used to generate the genome sequence (Rensing et al., 2008; Lang et al., 2018). However, over the years of cultivation in the lab, it has shown an accumulation of somatic mutations which was confirmed in this study and observed before, culminating in observable phenotypic changes (Meyberg et al., 2020). One of the characteristics of laboratory models is the capacity to maintain the organism cultivated in the lab for multiple generations, being able to progress through the complete life cycle. The reduction of fertility of Gd accessions in the lab limits experimental design, especially when studying sexual reproduction or when the generation of off-spring is required for the experiments. For this reason, the accession Reute, which shows the lowest number of differences with the Gd genome reference, and which has a much higher fertility than Gd (Meyberg et al., 2020) has been proposed as an alternative to study sexual reproduction (Hiss et al., 2017; Meyberg et al., 2020).

Due to changes in land use, at the original Gransden collection site no *P. patens* can be found anymore. However, phenotypic data suggest that Gransden was not always infertile, because Gd_JP shows intermediate fertility between Re and extant Gd_DE pedigrees (Hiss et al., 2017; Meyberg et al., 2020). Our data show that, as expected, Gd_UK shows the lowest number of SNPs as compared to the reference genome that was derived from Gd_2004 (UK). All other pedigrees show substantial and unique SNPs (Figure 5 and Supplementary Figure S11), demonstrating that during *in vitro* culture somatic mutations occur and accumulate in independent fashion. The practice of regular sexual reproduction of the cultured strains has the advantage that by this procedure it is ensured that the full life cycle can be followed. On top of that there is evidence that even during selfing *P. patens* is able to effectively purge deleterious mutations (Szövényi et al., 2017).

By comparing the normalized gene space SNP count of the Wi natural population samples with those of selfed progressions of Re and Gd laboratory strains we can estimate the genetic variability occurring in natural vs. laboratory samples (Figure 7 and Supplementary Figure S18). Interestingly, the variation of three generations of homozygous (selfed) Re and Gd offspring is similar to that observed in naturally occurring Wi samples (representing the same generation but four spore capsules and five spores each). Based on the normalized data, the three generations of selfed laboratory cultures might even have acquired and retained slightly more mutations than visible in the single Wi natural population. We conclude that a substantial amount of genetic variation occurs both through somatic mutation during vegetative propagation (Meyberg et al., 2020) as well as during sexual propagation by selfing. However, since the practice of regular selfing selects for fertility it seems preferable to follow that practice over exclusive vegetative propagation.

Like for the accessions, specific SNPs for each pedigree were extracted. The diversity of Gd pedigrees is lower than that of the accessions and hence there were not enough samples supporting the same SNP. To detect exclusive SNPs for each pedigree ranking the SNPs by sample support gave us the opportunity to extract the SNPs supported by most of the samples. Obligatory for this method is a correct metadata grouping of the samples. If samples would be described to be the wrong pedigree, exclusive SNPs cannot be accurately determined. Another issue is the sub-clustering of samples. We can observe this for the Gd_JP pedigree as well as for Gd_UK. There are SNPs in the Japan pedigree that occurred in St. Louis, after it was brought to the USA. Our Gd_JP sample set is mostly represented by samples from the USA. Extracted exclusive SNPs with high sample support can thus be scored for the JP- > USA pedigree, but maybe not for the full Gd_JP pedigree. Nevertheless, our provided exclusive SNP list can be used to classify the origin of unknown samples (Supplementary Figure S17).

### Experimental Confirmation of Selected SNPs

In large experiments that handle many samples, mistakes might occur during the management of the samples in the lab, in the sequencing facility or during later data analysis. The identification of exclusive SNPs in the *P. patens* accessions allows the detection and correction of mistakes in experimental metadata, such as the ones mentioned earlier (Figure 3), *in silico*. Moreover, the exclusive SNPs found in the different accessions were used to identify unique targets for restriction enzymes, allowing the development of RFLP assays to differentiate between the *P. patens* accessions. The presence of the predicted SNPs in all tested sequences confirms the successful and stringent SNP selection presented here. The successful establishment of the RFLP analysis for the *P. patens* accessions provides a fast and cheap tool to test the accession background of laboratory strains as well as newly collected *P. patens* accessions. With regard to the Gd pedigrees so far, no SNPs within a restriction enzyme site with enough sample coverage could be identified. However, differentiation between Gd_DE, Gd_JP and Gd_CH could be performed successfully based on the sequencing data (Supplementary Figure S17). Thus, SNPs between the Gd pedigrees need to be analyzed via sequencing so far, but including more Gd data sets in the presented approach and/or analyzing a small subset of Gd pedigrees could help to improve and identify SNPs, which could be used within a future RFLP approach to differentiate Gd pedigrees.

Independent of the RFLP method, the origin of *P. patens* plant material can be discovered by using the presented primers (Supplementary Tables S7, S8) and sequencing the amplicon. If sequencing data is already available (single fragments, RNA-seq or gDNA sample[s]), our pipeline and the exclusive SNP sets can be used to easily identify plant origins.

### Natural Population Variation and Selection

The number of observable mutations on the level of a naturally occurring population (Wi single spore isolates) is in the approximate same range as the mutations occurring in culture undergoing annual sexual reproduction (Figure 7 and Supplementary Figure S18). For samples mainly propagated vegetatively, observed mutations are somatic in nature. For samples that regularly go through sexual reproduction, changes introduced via meiotic recombination cannot be distinguished from somatic changes. Intriguingly, the number of detected SNPs was found to decline over time in samples with a known heritage of regular sexual reproduction (Supplementary Tables S12, S13). We take this as evidence that sexual reproduction, even in a haploid, selfing species is able to efficiently purge deleterious mutations, as previously shown (Szövényi et al., 2017).

Consequently, the majority of the observed mutations probably are somatic. The observed mutation rates (changes per year and site) are in the range of 7E-07 to 4E-06 (Supplementary Table S13). Studies in other plants found rates in the E-08 range (Hanlon et al., 2019; Schoen and Schultz, 2019). The observed *P. patens* mutation rates are approximately two orders of magnitude higher than the estimated rate of synonymous substitutions per synonymous site per year, 9E-09 (Rensing et al., 2007). Hence, *in vitro* propagation of *P. patens* apparently leads to the fixation of a higher number of mutations than occur naturally, and maybe more than described in other plant propagation systems. Many labs perform regular shredding of protonemal tissue for propagation. This mode of propagation might increase the number of fixed somatic mutations via induction of the DNA repair system through cell damage, potentially resulting in higher mutational load.

The Ka/Ks ratio of the Gransden pedigree generally is below 1, suggesting potential negative (purifying) selection on many loci (Supplementary Table S11). All other accessions, to the contrary, exhibit ratios larger than 1, suggesting potential positive (Darwinian) selection. The latter is regardless of whether they are naturally occurring (Wi) or cultured (Ka, Re, Vx). Potentially, the decades-long vegetative culture of Gd, most of it vegetatively, led to the expression of negative selection. All other accessions are much more recent isolates and in particular all Re samples studied went through annual sexual reproduction, which apparently effectively purges deleterious mutations. Interestingly, the GO terms over-represented among those genes affected by non-synonymous changes (Supplementary Figure S20) include microtubule-based movement (Re) and reproduction (Vx), fitting recently published data that show these terms contrasted between male infertile Gd and fertile Re (Meyberg et al., 2020). It appears probable that the artificial environment of vegetative *in vitro* Gd propagation led to a loss of fertility due to loss of selection pressure on genes required for sexual reproduction.

## Conclusion

Our study of sequence variants in *P. patens* laboratory strains revealed the accumulation of somatic mutations over years of cultivation, some of which can be detrimental e.g., with regard to fertility. It appears to be good practice to regularly let the lab cultures reproduce sexually, in order to keep selective pressure and to purge deleterious mutations. Since the original Gd accession is not available any more, and Gd JP shows less fertility than Re, it appears sensible to use Re (with its low number of SNPs as compared to Gd) for any studies that shall involve the life cycle. The identification of exclusive sets of SNPs for *P. patens* laboratory strains and accessions allowed the development of RFLP tests to identify the different accessions. Similarly, Gd pedigrees can be identified by sequencing of PCR products based on the pedigree-exclusive SNPs determined in this study. The variation of selfed laboratory strains is on the same order of magnitude as that of a natural population analyzed.

## Data Availability Statement

All RNA-seq samples used in this study are available via the NCBI SRA. Please see Supplementary Table S1 in Supplementary.pdf for more details.

## Author Contributions

FH analyzed the raw read data and performed SNP calling. DS-M, JL, P-FP, and RM contributed RNA-seq data. FH, NS, and RM setup and performed RFLP as well as sequencing analyses. SR conceived of the study and supervised it together with NF-P and P-FP. FH, NF-P, RM, and SR wrote the manuscript with the help of all authors.

## Conflict of Interest

The authors declare that the research was conducted in the absence of any commercial or financial relationships that could be construed as a potential conflict of interest.

## Footnotes

1 https://gatkforums.broadinstitute.org/gatk/discussion/3892/the-gatk-best-practices-for-variant-calling-on-rnaseq-in-full-detail

2 https://gatk.broadinstitute.org/hc/en-us/articles/360035531192-RNAseq-short-variant-discovery-SNPs-Indels

3 http://emboss.sourceforge.net/apps/cvs/emboss/apps/restrict.html

4 http://emboss.sourceforge.net/apps/cvs/emboss/apps/rebaseextract.html

5 https://bioinfogp.cnb.csic.es/tools/venny/index.html

## References

[B1] Ashton N. W. Cove D. J. (1977). The isolation and preliminary characterisation of auxotrophic and analogue resistant mutants of the moss, *Physcomitrella patens*. *Mol. Gen. Genet.* 154 87–95. 10.1007/bf00265581

[B2] Ashton N. W. Raju M. V. S. (2000). The distribution of gametangia on gametophores of *Physcomitrella* (Aphanoregma) patens in culture. *J. Bryol.* 22 9–12. 10.1179/jbr.2000.22.1.9

[B3] Beike A. K. von Stackelberg M. Schallenberg-Rüdinger M. Hanke S. T. Follo M. Quandt D. (2014). Molecular evidence for convergent evolution and allopolyploid speciation within the *Physcomitrium*-Physcomitrellaspecies complex. *BMC Evol. Biol.* 14:158. 10.1186/1471-2148-14-158 25015729 PMC4227049

[B4] Benjamini Y. Hochberg Y. (1995). Controlling the false discovery rate: a practical and powerful approach to multiple testing. *J. R. Stat. Soc. Ser. B (Methodological).* 57 289–300. 10.1111/j.2517-6161.1995.tb02031.x

[B5] Bolger A. M. Lohse M. Usadel B. (2014). Trimmomatic: a flexible trimmer for Illumina sequence data. *Bioinformatics (Oxford, England).* 30 2114–2120. 10.1093/bioinformatics/btu170 24695404 PMC4103590

[B6] Botstein D. White R. L. Skolnick M. Davis R. W. (1980). Construction of a genetic linkage map in man using restriction fragment length polymorphisms. *Am. J. Hum. Genet.* 32 314–331. 6247908 PMC1686077

[B7] Bouckaert R. Heled J. Kühnert D. Vaughan T. Wu C.-H. Xie D. (2014). BEAST 2: a software platform for bayesian evolutionary analysis. *PLoS Comput. Biol.* 10:e1003537. 10.1371/journal.pcbi.1003537 24722319 PMC3985171

[B8] Cingolani P. Platts A. Wang le L. Coon M. Nguyen T. Wang L. (2012). A program for annotating and predicting the effects of single nucleotide polymorphisms, SnpEff: SNPs in the genome of *Drosophila* melanogaster strain w1118; iso-2; iso-3. *Fly* 6 80–92. 10.4161/fly.19695 22728672 PMC3679285

[B9] Conway J. R. Lex A. Gehlenborg N. (2017). UpSetR: an R package for the visualization of intersecting sets and their properties. *Bioinformatics (Oxford, England).* 33 2938–2940. 10.1093/bioinformatics/btx364 28645171 PMC5870712

[B10] Cove D. (2005). The moss *Physcomitrella patens*. *Annu. Rev. Genet.* 39 339–358. 16285864 10.1146/annurev.genet.39.073003.110214

[B11] Cove D. J. Perroud P. F. Charron A. J. McDaniel S. F. Khandelwal A. Quatrano R. S. (2009). Isolation of DNA, RNA, and protein from the moss *Physcomitrella patens* gametophytes. *Cold Spring Harb. Protoc.* 2009:db.rot5146. 10.1101/pdb.prot5146 20147076

[B12] de Vries J. Rensing S. A. (2020). Gene gains paved the path to land. *Nat. Plants* 6 7–8. 10.1038/s41477-019-0579-5 31932675 PMC7116226

[B13] Demko V. Perroud P.-F. Johansen W. Delwiche C. F. Cooper E. D. Remme P. (2014). Genetic analysis of DEFECTIVE KERNEL1 loop function in three-dimensional body patterning in *Physcomitrella patens*. *Plant Physiol.* 166 903–919. 10.1104/pp.114.243758 25185121 PMC4213117

[B14] Ding X. Pervere L. M. Bascom C. Jr. Bibeau J. P. Khurana S. Butt A. M. (2018). Conditional genetic screen in *Physcomitrella patens* reveals a novel microtubule depolymerizing-end-tracking protein. *PLoS Genet.* 14:e1007221. 10.1371/journal.pgen.1007221 29746462 PMC5944918

[B15] Engel P. P. (1968). The induction of biochemical and morphological mutants in the moss *Physcomitrella patens*. *Am. J. Bot.* 55 438–446. 10.1002/j.1537-2197.1968.tb07397.x

[B16] Fernandez-Pozo N. Haas F. B. Meyberg R. Ullrich K. K. Hiss M. Perroud P.-F. (2019). PEATmoss (*Physcomitrella* expression atlas tool): a unified gene expression atlas for the model plant *Physcomitrella patens*. *Plant J.* 102 165–177. 10.1111/tpj.14607 31714620

[B17] Flowers J. M. Hazzouri K. M. Pham G. M. Rosas U. Bahmani T. Khraiwesh B. (2015). Whole-genome resequencing reveals extensive natural variation in the model green Alga *Chlamydomonas reinhardtii*. *Plant Cell* 27 2353–2369. 10.1105/tpc.15.00492 26392080 PMC4815094

[B18] Frank M. H. Scanlon M. J. (2015). Cell-specific transcriptomic analyses of three-dimensional shoot development in the moss *Physcomitrella patens*. *Plant J.* 83 743–751. 10.1111/tpj.12928 26123849

[B19] Hanlon V. C. T. Otto S. P. Aitken S. N. (2019). Somatic mutations substantially increase the per-generation mutation rate in the conifer *Picea sitchensis*. *Evol. Lett.* 3 348–358. 10.1002/evl3.121 31388445 PMC6675141

[B20] Hiss M. Meyberg R. Westermann J. Haas F. B. Schneider L. Schallenberg-Rdinger M. (2017). Sexual reproduction, sporophyte development and molecular variation in the model moss *Physcomitrella patens*: introducing the ecotype Reute. *Plant J. Cell Mol. Biol.* 90 606–620. 10.1111/tpj.13501 28161906

[B21] Huson D. H. Bryant D. (2005). Application of phylogenetic networks in evolutionary studies. *Mol. Biol. Evol.* 23 254–267. 10.1093/molbev/msj030 16221896

[B22] Kamisugi Y. Von Stackelberg M. Lang D. Care M. Reski R. Rensing S. A. (2008). A sequence-anchored genetic linkage map for the moss, *Physcomitrella patens*. *Plant J.* 56 855–866. 10.1111/j.1365-313x.2008.03637.x 18657236 PMC2667646

[B23] Krasovec M. Eyre-Walker A. Sanchez-Ferandin S. Piganeau G. (2017). Spontaneous mutation rate in the smallest photosynthetic eukaryotes. *Mol. Biol. Evol.* 34 1770–1779. 10.1093/molbev/msx119 28379581 PMC5455958

[B24] Lang D. Ullrich K. K. Murat F. Fuchs J. Jenkins J. Haas F. B. (2018). The *Physcomitrella patens* chromosome-scale assembly reveals moss genome structure and evolution. *Plant J. Cell Mol. Biol.* 93 515–533. 10.1111/tpj.13801 29237241

[B25] Leaché A. D. Oaks J. R. (2017). The utility of single nucleotide polymorphism (SNP) data in phylogenetics. *Annu. Rev. Ecol. Evol. Syst.* 48 69–84. 10.1146/annurev-ecolsys-110316-022645

[B26] Li H. Handsaker B. Wysoker A. Fennell T. Ruan J. Homer N. (2009). The sequence alignment/map format and SAMtools. *Bioinformatics* 25 2078–2079. 10.1093/bioinformatics/btp352 19505943 PMC2723002

[B27] Ma X. Shao Y. Tian L. Flasch D. A. Mulder H. L. Edmonson M. N. (2019). Analysis of error profiles in deep next-generation sequencing data. *Genome Biol.* 20:50. 10.1186/s13059-019-1659-6 PMC6417284 30867008

[B28] McKenna A. Hanna M. Banks E. Sivachenko A. Cibulskis K. Kernytsky A. (2010). The genome analysis toolkit: a MapReduce framework for analyzing next-generation DNA sequencing data. *Genome Res.* 20 1297–1303. 10.1101/gr.107524.110 20644199 PMC2928508

[B29] Medina R. Johnson M. G. Liu Y. Wickett N. J. Shaw A. J. Goffinet B. (2019). Phylogenomic delineation of *Physcomitrium* (Bryophyta: Funariaceae) based on targeted sequencing of nuclear exons and their flanking regions rejects the retention of *Physcomitrella*, *Physcomitridium* and *Aphanorrhegma*. *J. Syst. Evol.* 57 404–417. 10.1111/jse.12516

[B30] Meyberg R. Perroud P.-F. Haas F. B. Schneider L. Heimerl T. Renzaglia K. S. (2020). Characterization of evolutionarily conserved key players affecting eukaryotic flagellar motility and fertility using a moss model. *New Phytol.* 10.1111/nph.16486 [Epub ahead of print]. 32064607 PMC8224819

[B31] Moody L. A. Kelly S. Rabbinowitsch E. Langdale J. A. (2018). Genetic regulation of the 2D to 3D growth transition in the moss *Physcomitrella patens*. *Curr. Biol.* 28 473–478.e5. 10.1016/j.cub.2017.12.052 29395927 PMC5807088

[B32] Nguyen T.-P. Muhlich C. Mohammadin S. van den Bergh E. Platts A. E. Haas F. B. (2019). Genome improvement and genetic map construction for aethionema arabicum, the first divergent branch in the Brassicaceae family. *G3 (Bethesda, Md).* 9 3521–3530. 10.1534/g3.119.400657 31554715 PMC6829135

[B33] Niu S. Song Q. Koiwa H. Qiao D. Zhao D. Chen Z. (2019). Genetic diversity, linkage disequilibrium, and population structure analysis of the tea plant (*Camellia sinensis*) from an origin center, Guizhou plateau, using genome-wide SNPs developed by genotyping-by-sequencing. *BMC Plant Biol.* 19:328. 10.1186/s12870-019-1917-5 31337341 PMC6652003

[B34] Perroud P.-F. Cove D. J. Quatrano R. S. McDaniel S. F. (2011). An experimental method to facilitate the identification of hybrid sporophytes in the moss *Physcomitrella patens* using fluorescent tagged lines. *New Phytol.* 191 301–306. 10.1111/j.1469-8137.2011.03668.x 21366596 PMC3445409

[B35] Perroud P.-F. Haas F. B. Hiss M. Ullrich K. K. Alboresi A. Amirebrahimi M. (2018). The *Physcomitrella patens* gene atlas project: large scale RNA-seq based expression data. *Plant J.* 95 168–182. 10.1111/tpj.13940 29681058

[B36] Perroud P.-F. Meyberg R. Rensing S. A. (2019). *Physcomitrella patens* reute mCherry as a tool for efficient crossing within and between ecotypes. *Plant Biol.* 21 143–149. 10.1111/plb.12840 29772086

[B37] Quinlan A. R. Hall I. M. (2010). BEDTools: a flexible suite of utilities for comparing genomic features. *Bioinformatics (Oxford, England).* 26 841–842. 10.1093/bioinformatics/btq033 20110278 PMC2832824

[B38] Rensing S. A. (2018). Great moments in evolution: the conquest of land by plants. *Curr. Opin. Plant Biol.* 42 49–54. 10.1016/j.pbi.2018.02.006 29525128

[B39] Rensing S. A. Beike A. K. Lang D. (2013). “Evolutionary importance of generative polyploidy for genome evolution of haploid-dominant land plants,” in *Plant Genome Diversity : Physical Structure, Behaviour and Evolution of Plant Genomes*, Vol. 2 eds Leitch I. J. Greilhuber J. Jaroslav D. Jonathan W. (Vienna: Springer-Verlag), 295–305. 10.1007/978-3-7091-1160-4_18

[B40] Rensing S. A. Goffinet B. Meyberg R. Wu S.-Z. Bezanilla M. (2020). The moss *Physcomitrium* (*Physcomitrella*) *patens*: a model organism for non-seed plants. *Plant Cell* 32 1361–1376. 10.1105/tpc.19.00828 32152187 PMC7203925

[B41] Rensing S. A. Ick J. Fawcett J. A. Lang D. Zimmer A. Van de Peer Y. (2007). An ancient genome duplication contributed to the abundance of metabolic genes in the moss *Physcomitrella patens*. *BMC Evol. Biol.* 7:130. 10.1186/1471-2148-7-130 17683536 PMC1952061

[B42] Rensing S. A. Lang D. Zimmer A. D. Terry A. Salamov A. Shapiro H. (2008). The *Physcomitrella* genome reveals evolutionary insights into the conquest of land by plants. *Science* 319 64–69. 18079367 10.1126/science.1150646

[B43] Ribeiro A. Golicz A. Hackett C. A. Milne I. Stephen G. Marshall D. (2015). An investigation of causes of false positive single nucleotide polymorphisms using simulated reads from a small eukaryote genome. *BMC Bioinformatics* 16:382. 10.1186/s12859-015-0801-z 26558718 PMC4642669

[B44] Saint-Marcoux D. Billoud B. Langdale J. A. Charrier B. (2015). Laser capture microdissection in *Ectocarpus siliculosus*: the pathway to cell-specific transcriptomics in brown algae. *Front. Plant Sci.* 6:54. 10.3389/fpls.2015.00054 25713580 PMC4322613

[B45] Schmieder R. Edwards R. (2011). Quality control and preprocessing of metagenomic datasets. *Bioinformatics* 27 863–864. 10.1093/bioinformatics/btr026 21278185 PMC3051327

[B46] Schoen D. J. Schultz S. T. (2019). Somatic mutation and evolution in plants. *Annu. Rev. Ecol. Evol. Syst.* 50 49–73. 10.1146/annurev-ecolsys-110218-024955

[B47] Schween G. Schulte J. Reski R. Hohe A. (2005). Effect of ploidy level on growth, differentiation, and morphology in *Physcomitrella patens*. *Bryologist.* 108 27–35. 10.1639/0007-2745(2005)108[27:eoplog]2.0.co;2

[B48] Smits W. K. (2017). SNP-ing out the differences: investigating differences between *Clostridium* difficile lab strains. *Virulence* 8 613–617. 10.1080/21505594.2016.1250998 27791481 PMC5626201

[B49] Stevenson S. R. Kamisugi Y. Trinh C. H. Schmutz J. Jenkins J. W. Grimwood J. (2016). Genetic analysis of *Physcomitrella patens* identifies Abscisic acid non-responsive, a Regulator of ABA responses unique to basal land plants and required for desiccation tolerance. *Plant Cell* 28 1310–1327. 27194706 10.1105/tpc.16.00091 PMC4944411

[B50] Szövényi P. Ullrich K. K. Rensing S. A. Lang D. van Gessel N. Stenøien H. K. (2017). Selfing in haploid plants and efficacy of selection: codon usage bias in the model moss *Physcomitrella patens*. *Genome Biol. Evol.* 9 1528–1546. 10.1093/gbe/evx098 28549175 PMC5507605

[B51] Vashisht D. Hesselink A. Pierik R. Ammerlaan J. M. Bailey-Serres J. Visser E. J. (2011). Natural variation of submergence tolerance among *Arabidopsis thaliana* accessions. *New Phytol.* 190 299–310. 10.1111/j.1469-8137.2010.03552.x 21108648

[B52] von Stackelberg M. Rensing S. A. Reski R. (2006). Identification of genic moss SSR markers and a comparative analysis of twenty-four algal and plant gene indices reveal species-specific rather than group-specific characteristics of microsatellites. *BMC Plant Biol.* 6:9. 10.1186/1471-2229-6-9 16734891 PMC1526434

[B53] Wang S. Meyer E. McKay J. K. Matz M. V. (2012). 2b-RAD: a simple and flexible method for genome-wide genotyping. *Nat. Methods* 9 808–810. 10.1038/nmeth.2023 22609625

[B54] Widiez T. Symeonidi A. Luo C. Lam E. Lawton M. Rensing S. A. (2014). The chromatin landscape of the moss *Physcomitrella patens* and its dynamics during development and drought stress. *Plant J.* 79 67–81. 10.1111/tpj.12542 24779858

[B55] Wu T. D. Nacu S. (2010). Fast and SNP-tolerant detection of complex variants and splicing in short reads. *Bioinformatics* 26 873–881. 10.1093/bioinformatics/btq057 20147302 PMC2844994

[B56] Xia W. Luo T. Zhang W. Mason A. S. Huang D. Huang X. (2019). Development of high-density SNP markers and their application in evaluating genetic diversity and population structure in *Elaeis guineensis*. *Front. Plant Sci.* 10:130. 10.3389/fpls.2019.00130 30809240 PMC6380268

