# Supplementary material for: Single Nucleotide Polymorphism Charting of P. patens Reveals Accumulation of Somatic Mutations During in vitro Culture on the Scale of Natural Variation by Selfing
Source: Front Plant Sci. 2020 Jul 7;11:813. doi: 10.3389/fpls.2020.00813 (PMC7358436; doi:10.3389/fpls.2020.00813)
Supplement: Supplementary file 1 [file Presentation_1.zip › 536410_Haas_et_al_Supplement/Supplementary.zip!/Supplementary.pdf]

Supplementary data for Haas et al.

Table S1: Source and NCBI accession nos. for all RNA-seq samples used.

<table>
<tr><th>Accession</th><th>Project/tissue</th><th>Source</th><th>BioProject/SRA number</th></tr>
<tr><td rowspan="5">Gransden</td><td>JGI Gene Atlas</td><td>(Fernandez-Pozo et al. 2019, Perroud et al. 2018)</td><td>PRJNA259145, PRJNA259146, PRJNA259147, PRJNA373582, PRJNA373583, PRJNA373584, PRJNA373585, PRJNA373586, PRJNA373587, PRJNA373588, PRJNA373589, PRJNA373590, PRJNA373591, PRJNA373592, PRJNA373593, PRJNA373594, PRJNA373595, PRJNA373596, PRJNA373598, PRJNA411163 [b], PRJNA411164, PRJNA411165, PRJNA411166, PRJNA411167, PRJNA411168, PRJNA411169, PRJNA411170, PRJNA411171, PRJNA411172, PRJNA411173, PRJNA411174, PRJNA411175, PRJNA411176, PRJNA411177, PRJNA411178, PRJNA411179, PRJNA411180, PRJNA411181, PRJNA411182, PRJNA411202, PRJNA411203, PRJNA411204, PRJNA411205, PRJNA411206, PRJNA411207, PRJNA411208, PRJNA411209, PRJNA411210, PRJNA411211, PRJNA411212, PRJNA411213, PRJNA411214, PRJNA411215, PRJNA411216</td></tr>
<tr><td>DEK1</td><td>(Demko et al. 2014)</td><td>PRJEB6339</td></tr>
<tr><td>ABA response</td><td>(Stevenson et al. 2016)</td><td>SRX1176825, SRX1176826</td></tr>
<tr><td>Shoot development</td><td>(Frank and Scanlon 2015)</td><td>SRX803263, SRX803262, SRX803261, SRX803260, SRX803259, SRX803257, SRX682830, SRX682829, SRX682828, SRX682827, SRX682822, SRX682821</td></tr>
<tr><td>Antheridia bundles</td><td>(Meyberg et al. 2020)</td><td>PRJNA559055</td></tr>
</table>

**Kaskaskia**	Protonema	This study	PRJNA601618

**Reute**	JGI Gene Atlas	(Fernandez-Pozo et al. 2019, Perroud et al. 2018)	PRJNA259147, PRJNA411184, PRJNA411185, PRJNA411186, PRJNA411187, PRJNA411188, PRJNA411189, PRJNA411190, PRJNA411191, PRJNA411192, PRJNA411193, PRJNA411194, PRJNA411195, PRJNA411196, PRJNA411197, PRJNA411198, PRJNA411199, PRJNA411200, PRJNA411201
	Antheridia bundles	(Meyberg et al. 2020)	PRJNA559055
	Sporophytic tissue, ES1	This study	PRJNA600210

**Villersexel**	Laser capture of sexual reproduction stages	This study	PRJNA602303
	Codon Usage Bias	(Szövényi et al. 2017)	PRJEB19978
**Wisconsin**	gDNA Single spores	This study	PRJNA602210

[b] Sample meta data are defining samples as accession Gransden, however, this study suggests that they are from accession Kaskaskia.

Table S2: *P. patens* Kaskaskia RNA-seq experiment description and accession number.

	**Tissue**	**Medium**	**Treatment**	**SRA**
K-I	Protonema, gametophore and sporophyte, 7 days and 3 months	BCD	Mixed:<br>1/3: protonema with 1 hour ABA 5µM, 5 NAA 5µM, BAP µM, 8°C treatments<br>1/3: ABA 5µM, 5 NAA 5µM, BAP µM, 8°C treatments<br>1/3: Three months gametophores with sporophytes	SRR10902189
K-II	Protonema, 7 day	BCD<br>+ 5mM glucose	Long day light condition	SRR10902188
K-III	Protonema, 7 day	BCD<br>+ 5mM glucose	No light	SRR10902187
K-IV	Protonema, 7 day	BCD	Harvest at 08h00 (dawn)	SRR10902186
K-V	Protonema, 7 day	BCD	Harvest at 12h00	SRR10902185
K-VI	Protonema, 7 day	BCD	Harvest at 16h00	SRR10902184
K-VII	Protonema, 7 day	BCD	Harvest at 20h00	SRR10902183
K-VIII	Protonema, 7 day	BCD<br>+ 5 mM Ammonium tartrate	6 days standard +<br>24h Glyphoste 5 mM	SRR10902182
K-IX	Protonema, 7 day	BCD	Long day light condition	SRR10902181
K-X	Protonema, 7 day	BCD<br>+5 mM Ammonium tartrate	Long day light condition	SRR10902180

Table S3: Read distribution.

Accession	Project	Raw data	Uniquely mapped reads	used for GATK pipeline
Gransden	**JGI Gene Atlas**	2,803,540,901	2,275,015,526	1,986,071,695
	**DEK1**	142,060,456	117,626,868	57,701,716
	**ABA response**	71,105,859	46,327,636	12,673,554
	**Shoot development**	133,665,970	101,707,266	37,057,354
	**Antheridia bundles**	114,584,205	85,684,460	47,769,995
Kaskaskia	**Protonema**	650,738,592	541,188,206	420,973,321
Reute	**JGI Gene Atlas**	525,465,788	419,522,685	374,992,382
	**Antheridia bundles**	115,771,691	84,302,632	46,638,828
	**Sporophytic tissue**	39,203,989	33,804,665	9,340,399
Villersexel	**Laser capture of sexual reproduction stages**	19,569,737	13,820,142	7,105,192
	**Codon Usage Bias**	68,390,324	59,697,452	30,875,300
Wisconsin	**gDNA single spores**	1,062,163,031	845,313,241	473,057,571
	**Sum (RNA-seq)**	4,684,097,512	3,778,697,538	3,031,199,736
	**Sum (gDNA)**	1,062,163,031	845,313,241	473,057,571

Table S4: RNA-seq read distribution for each accession

	Raw reads	After read filtering	Reads for SNP calling
Gransden	68%	68%	70%
Kaskaskia	12%	13%	14%
Reute	18%	16%	15%
Villersexel	2%	2%	1%

Table S5: Number of intersected RNA-seq SNPs to the gDNA SNPs from Lang et al. 2018

	vcf [a]	cov_filter [b]	support_3 [c]	wo_indels [d]
Reute	20,221 (9%)	12,911 (13%)	12,601 (14%)	10,988 (26%)
Villersexel	220,663 (54%)	65,884 (82%)	50,528 (86%)	47,031 (89%)
Kaskaskia	32,307 (9%)	23,844 (15%)	23,779 (17%)	21,644 (28%)

The percentage values in brackets are representing the proportion of RNA-seq SNPs found in the gDNA SNP set. [a] Number of raw SNPs called by the GATK pipeline. [b] Number of RNA-seq SNPs filtered by read coverage, [c] plus minimum three samples need to support the SNP and [d] in addition to the filter steps before, the indels were removed.

Table S6: SNP normalization methods.

Accession	Pedigree	Sample	SNP / covered bp	SNP / number of reads	SNP / number of genes	SNP / gene length [bp]	covered bp / SNP
**Gd**			0.00034	0.00094	1.206	0.00040	4,666
	**Gd_DE**		0.00038	0.00113	-	-	2,871
	**Gd_UK**		0.00028	0.00090	-	-	6,206
		**2004**	0.00006	0.00009	-	-	15,512
		**Birmingham**	0.00035	0.00117	-	-	3,104
	**Gd_CH**		0.00042	0.00105	-	-	2,402
	**Gd_JP**		0.00022	0.00049	-	-	9,526
		**St. Lousi PE**	0.00044	0.00102	-	-	2,284
		**St. Lousi SE**	0.00007	0.00010	-	-	15,324
		**Okazaki & Columbia**	0.00038	0.00090	-	-	2,756
**Ka**			0.00159	0.00297	2.300	0.00076	630
**Re**			0.00058	0.00178	1.280	0.00043	1,912
**Vx**			0.01761	0.01488	1.607	0.00053	143
**Wi**			0.06641	0.00363	1.925	0.00064	206

Table S7: SNPs selected for SNP verification via sequencing and RFLP analysis with their corresponding primer pairs, melting temperatures, expected amplicon length (nt), restriction enzymes and expected fragments after restriction (nt).

Accession	Position	SNP	Primer	Sequence	Tm (°C)	Amplicon (nt)	Enzyme	Restriction fragments (nt)
**Re**	Chr03:17,747,483	A→T	Re1_for	ACCGATCTCGTGGATAAGAG	59.9	1,255	NdeI	265 | 990
			Re1 _rev	TTCGAGGACTTGCAAAGG	58.8			
**Ka**	Chr01:25,061,888	C→A	Ka1 _for	GTTCTCACCATGAGGGAGTTAC	61.6	1,342	XbaI	984 | 358
			Ka1 _rev	GATGGACCCTGATTATAGCG	58.9			
**Vx**	Chr03:2,712,099	A→G	Vx1 _for	AGCTCAGAGTTCAATCACC	58.3	1,366	SwaI	1,063 | 303
			Vx1_rev	ACGCTGACGACTTACTCTAC	60.4			

Table S8: SNPs selected for SNP verification via sequencing.

Accession	SNP position	SNP	Primer	Sequence	Tm (°C)	Amplicon (nt)
**Re**	Chr04:21,933,417	G→C	Re2_for	GTTGTCCCAGATGGCAA	58,3	881
			Re2 _rev	TTGCAGTCTCGACTACTC	57,2	
**Vx**	Chr13:4,764,050	A→T	Vx2 _for	AAGGAGCAGGAGACTAAAC	57,6	1,128
			Vx2 _rev	CAAGTCCAGAAGTCCTCTAC	58,2	
**Gd_DE_MR**	Chr02:12,750,876	C→G	Gd_DE_1_for	GATCAGCTGGTTAGTGTTTGG	60.4	1,307
			Gd_DE_1_rev	GTCTTCAAGTGCTTGGCA	59.5	
**Gd_DE_MR**	Chr05:3,105,395	C→A	Gd_DE_2_for	AGCAGAAGGTTCAATGAGG	58.6	1,312
			Gd_DE_2_rev	TCTACGCTTAAAGGTCTCACG	61	
**Gd_DE_MR**	Chr12:2,095,061	A→T	Gd_DE_3_for	TGATTCGAATGTGCTGTGACG	62.8	1,283
			Gd_DE_3_rev	AGAAATGAACTCAAGGTGCGG	62.4	
**Gd_JP**	Chr20:8,688,243	G→T	Gd_JP_for	CAGCAATGGAAAGGTCTGGC	59	775
			Gd_JP_rev	AACATAGGCAAGCGAACCAG	59	
**Gd_CH**	Chr23:11,248,087	A→G	Gd_Ch_for	AAATCCGACATTTCCCAGCC	59	731
			Gd_Ch_rev	AACCCTACACTGTCGATGGC	59	

Table S9: List of sequencing files of amplicons for the selected SNPs within the different accessions and pedigrees.

**Accession**	**SNP position**	**Gd_DE_MR**	**Re**	**Ka**	**Vx**	**Gd_JP_Okazaki**	**Gd_JP_St.Louis**	**Gd_UK**	**Gd_CH**
**Re**	Chr03:17747483	1CEDZAB092	1CEDZAB093	1CEDZAB105	1CEDZAB095				
**Re**	Chr04:21933417	1CEDZAB096	1CEDZAB097	1CEDZAB098	1CEDZAB099				
**Ka**	Chr25:2762907	1CEDZAB084	1CEDZAB085	1CEDZAB086	1CEDZAB087				
**Vx**	Chr03:2712099	1CEDZAB079	1CEDZAB100	1CEDZAB080	1CEDZAB081				
**Vx**	Chr13:4764050	1CEDZAB088	1CEDZAB089	1CEDZAB090	1CEDZAB091				
**Gd_DE_MR**	Chr02:12750876	1CEDZAB156	1CEDZAB157	1CEDZAB160	1CEDZAB159	1CEDZAB158			
**Gd_DE_MR**	Chr05:3105395	1CEDZAB000	1CEDZAB167	1CEDZAB170	1CEDZAB169	1CEDZAB168	1CEDZAB001	1CEDZAB002	1CEDZAB003
**Gd_DE_MR**	Chr12:2095061	1CEDZAB173	1CEDZAB174	1CEDZAB177	1CEDZAB176	1CEDZAB175			
**Gd_JP**	Chr20:8688243	1CEDZAB008					1CEDZAB009	1CEDZAB0010	1CEDZAB011
**Gd_CH**	Chr13:4764050	1CEDZAB012					1CEDZAB013	1CEDZAB014	1CEDZAB015

Table S10: Ploidy of *P. patens* plants used for sequencing.

P. patens accession / pedigree	Ploidy tested	haploid?	Differences in GATK SNP calling 1n and 2n
Gd_Beijing-2010	N/A		63.4%
Gd_Berlin	yes	x	51.7%
Gd_Birmingham	no		47.3%
Gd_Birmingham-2009	no		61.5%
Gd_Columbia-2010	N/A		45.7%
Gd_FR-WT9	yes	x	56.9%
Gd_FR-WT11	yes	x	56.9%
Gd_Gransden-2004	yes	x	62.8%
Gd_Gransden-WT9	yes	x	55.2%
Gd_MR-WT11	no		56.0%
Gd_MR-WT12	yes	x	61.0%
Gd_MR-WT15	yes	Phenotypically 1n [a]	36.2%
Gd_Okazaki-1998	N/A		60.6%
Gd_Padova-2008	N/A		58.8%
Gd_St.Louis-2007	N/A		63.8%
Gd_St.Louis-2007_JGI	no	offspring phenotypically 1n [b]	58.9%
Gd_St.Louis	N/A		30.7%
Gd_Uruguay-WT9	yes	x	68.0%
Gd_Versaille	N/A		59.2%
Gd_WT-Grenoble	N/A		57.3%
Ka_kaskaskia	no	offspring phenotypically 1n [b]	81.1%
Re_Reute-2007	yes	x	67.7%
Re_Reute-2012	no		61.9%
Re_Reute-2015	yes	Phenotypically 1n [a]	59.4%
Vx_K3	no		90.7%
Vx_unknown	yes	offspring, allele-specific expression 1n	92.9%
Wi_2018	no		88.1%

[a] Plants were check by phenotypical observation (polyploid plants usually have a 'fluffy' habitus that haploid plants do not exhibit).

[b] Offspring of the used plants were checked by phenotypical observation.

The colours in the last column indicates the differences of the GATK 1n and 2n runs.

Table S11: Total number of synonymous and non-synonymous SNPs.

**Accession/pedigree**	**Non-synonymous**	involved genes	**Synonymous**	involved genes	**Ka/Ks**
**Gd**	62,142	579	69,870	421	0.889
Gd_FR-WT9 *	2,743	241	3,162	172	0.867
Gd_Uruguay-WT9 *	2,408	226	2,822	167	0.853
Gd_Gransden-WT9 *	8,308	276	9,440	198	0.880
Gd_FR-WT11 *	1,471	201	1,583	138	0.929
Gd_MR-WT11 *	6,415	291	6,840	208	0.938
Gd_MR-WT12 *	7,575	253	8,327	184	0.910
Gd_MR-WT15 *	1,143	213	1,631	145	0.701
**Re**	55,916	1725	39,416	1,065	1.419
Re_Reute-2007 *	10,063	1473	7,246	889	1.389
Re_Reute-2012 *	39,233	1,575	27,429	973	1.430
Re_Reute-2015 *	6,620	1,461	4,741	882	1.396
**Ka**	142,009	5,599	93,964	4,020	1.511
**Vx**	44,976	6,703	34,270	5,391	1.312
**Wi**	208,930	7,834	118,430	5,115	1.764

The sum of all detected synonymous and non-synonymous SNPs are shown. If a gene contains multiple effects, it was only counted once. The 99% confidence interval of the linear regression of Ka/Ks over all samples is 1.1..1.2, i.e. all samples shown here are above or below the confidence interval, suggesting negative (Gd) or positive selection (other pedigrees) (Supplementary File 8). * Pedigrees with known propagation history.

Table S12: Average number of SNPs (filtered by all three methods) per sample with documented propagation history.

***P. patens* accession / pedigree**	**Average SNP number**	**Source**
Gd_FR-WT9	6,452	Perroud et al. 2018
Gd_Uruguay-WT9	7,851	Perroud et al. 2018
Gd_Gransden-WT9	6,778	JGI_2nd_round
Gd_FR-WT11	7,244	Perroud et al. 2018
Gd_MR-WT11	7,271	Perroud et al. 2018
Gd_MR-WT12	6,221	JGI_2nd_round
Gd_MR-WT15	2,555	Meyberg et al. 2020
Re_Reute-2007	13,282	Perroud et al. 2018
Re_Reute-2012	11,927	JGI_2nd_round
Re_Reute-2015	8,635	Meyberg et al. 2020

In the first column, mutant or wildtype experiments are annotated. The colours in the middle column indicate the average number of SNPs called for that experiment (green represents high numbers, blue low numbers).

Table S13: Mutations per year and site for all pedigrees with a documented propagation history.

	Total SNPs [a]	Unique SNPs [b]	Mutations per year	Mutation rate [c]
WT9	31,230	3,745		
Gd_MR-WT11	26,525	1,483	742	1.571E-06
Gd_MR-WT12	23,557	642	642	1.361E-06
Gd_MR-WT15	3,757	1,047	349	7.396E-07
WT9	31,230	18,435		
FR-WT11	13,202	407	136	2.875E-07
Re_Reute-2007	27,267	3,327		
Re_Reute-2012	36,051	10,560	2112	4.476E-06
Re_Reute-2015	13,266	2,234	745	1.578E-06

[a] SNPs had to pass all three filter methods. [b] Unique SNPs derived from the intersection of the shown pedigrees (WT9 and Re 2007 are the respective ancestors). [c] The *P. patens* genome size is 471,852,792 bp.

Table S14: *P. patens* accession genotypes as detected by the GATK ploidy = 2 run.

	homozygous		heterozygous			
	1/1		0/1		1/2	
**Gd**	2,293,020	42.00%	3,165,085	57.98%	1,161	0.02%
**Re**	670,399	50.33%	659,948	49.55%	1,597	0.12%
**Vx**	629,961	86.03%	102,076	13.94%	255	0.03%
**Ka**	1,360,047	67.95%	639,305	31.94%	2,172	0.11%
**Wi**	25,863,758	82.26%	5,572,461	17.72%	6,662	0.02%

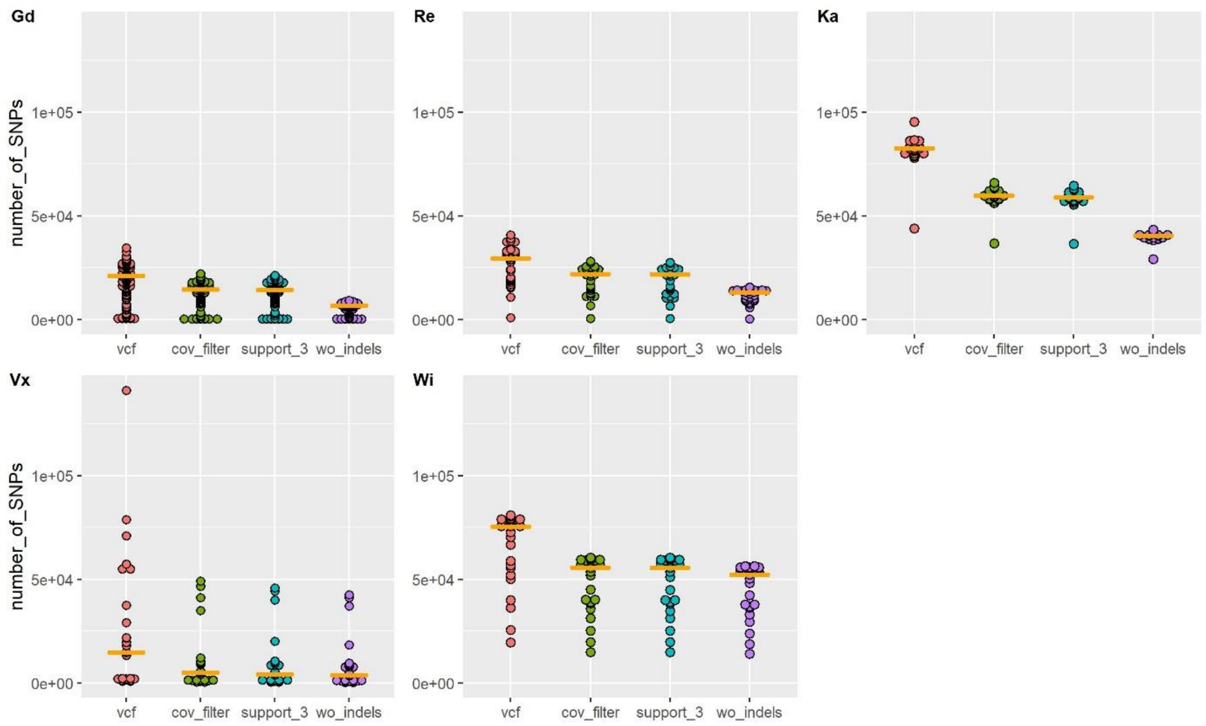

Figure S1: SNP reduction by filtering.

Each of the five plots shows the GATK SNP output on the left (no filter [vcf]) and the different filter steps applied to the data on the right (read coverage filter [cov_filter] and >= three samples supporting the SNP [support_3]). The strictest filter is rightmost (removing indels [wo_indels]). Each filter step is based on the remaining SNPs after applying the previous filter (for each accession left to right). Individual samples are represented by dots, the median by an orange horizontal bar. For details of filter steps refer to Materials and Methods.

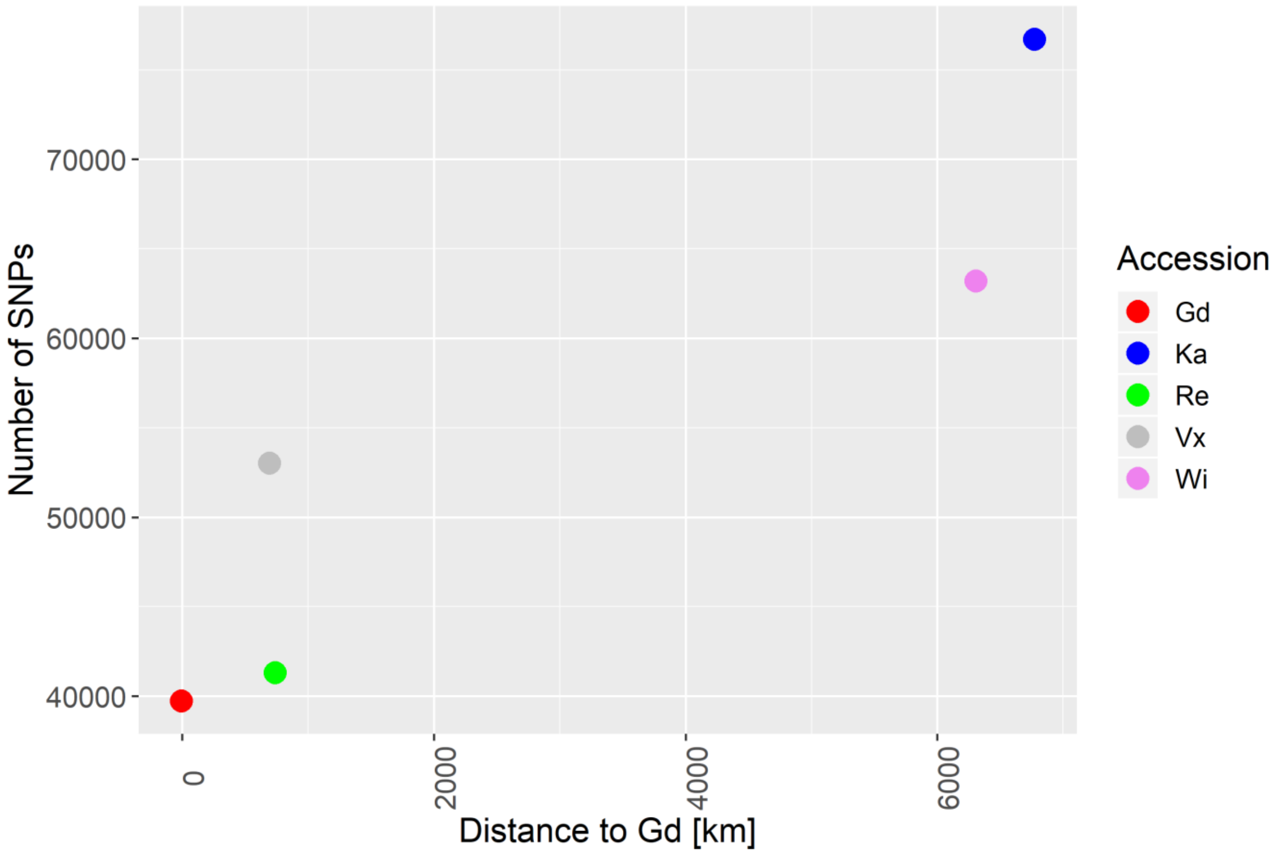

Figure S2: Number of SNPs applied to the line of sight distance to the reference Gransden.

Distance to Gd: Vx = 700 km; Re = 730 km, Wi = 6,300 km; Ka = 6,750 km.

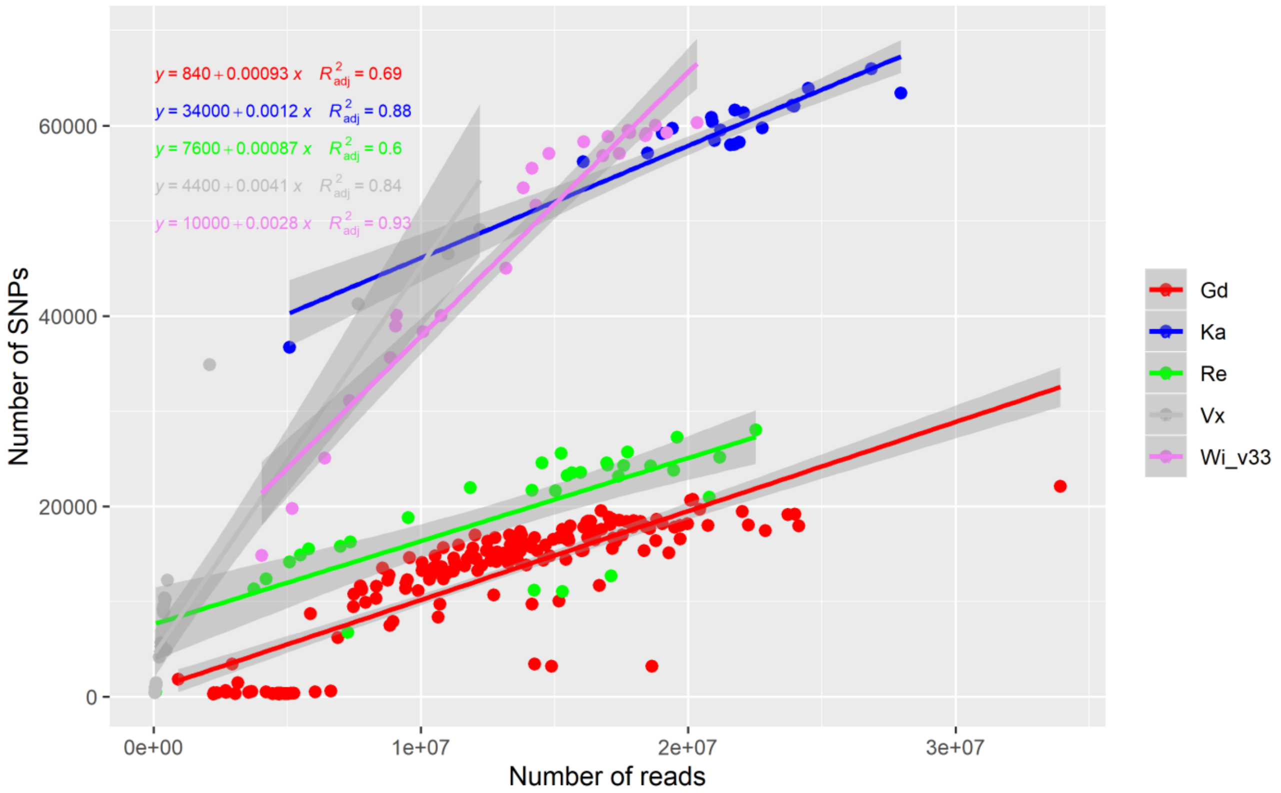

Figure S3: SNP normalization by read number.

Number of SNPs (filtered by read coverage, minimum support of three samples and removed indels) to the number of reads per sample. The slope is calculated by R method “lm” and in grey the confidence interval is shown around the regression lines.

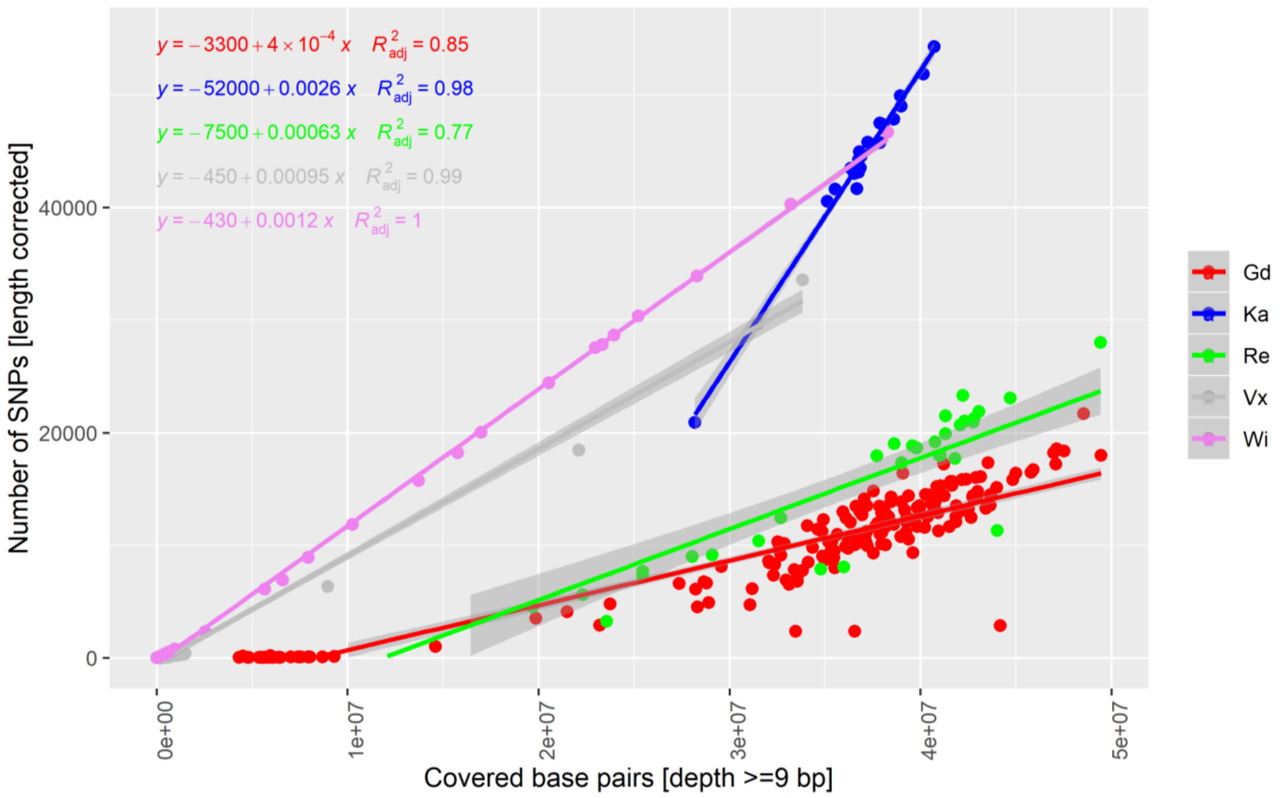

Figure S4: SNP normalization by covered base pairs.

Covered base pairs with read depth >= nine applied to the number of SNPs, filtered by read coverage, sample support of three and removed indels. The number of SNPs were corrected by the maximum number of covered base pairs. The slope was calculated by the R method "lm" and in grey the confidence interval is shown around the regression lines.

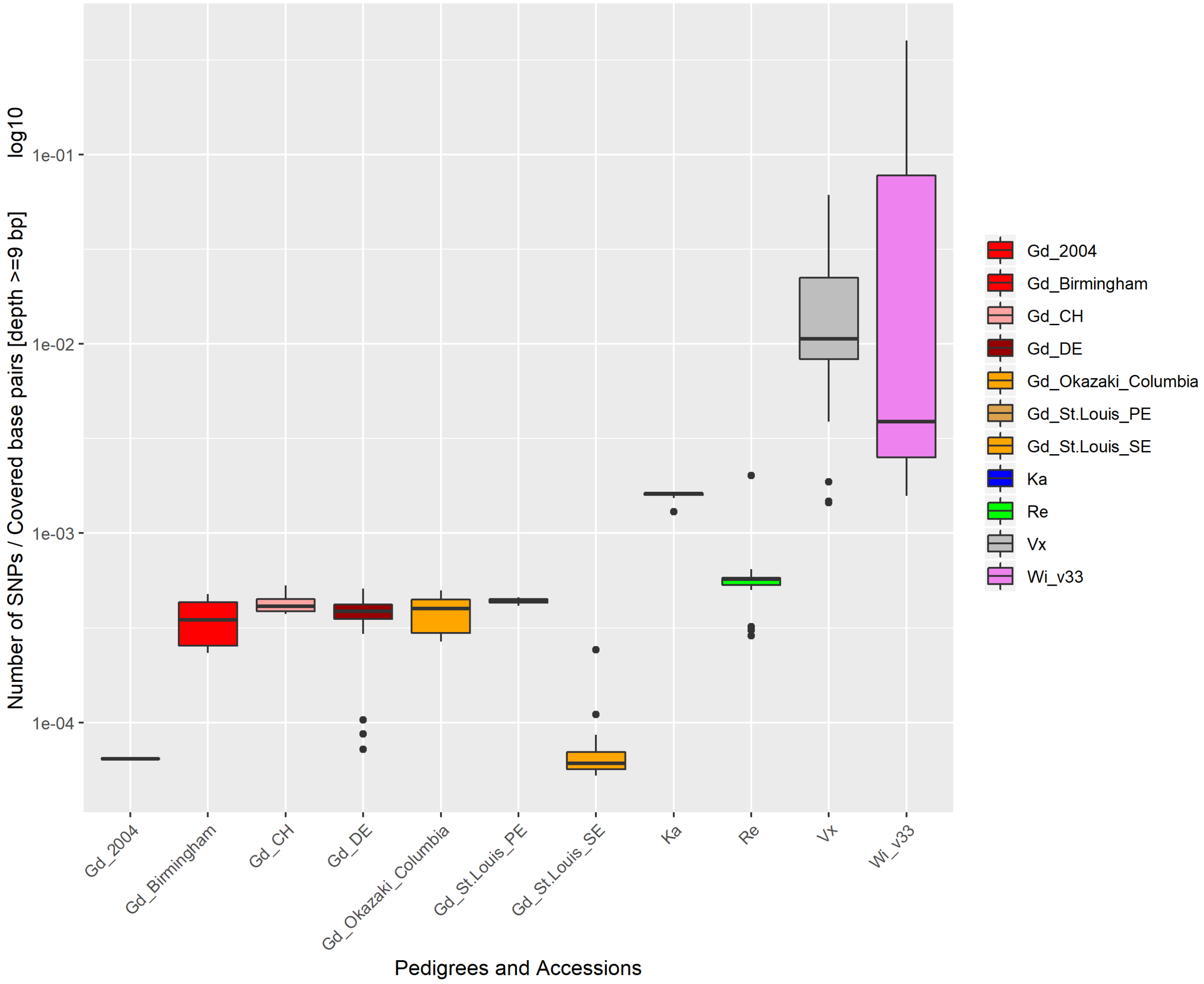

Figure S5: Number of SNPs divided by the number of covered base pairs.

The number of SNPs (three filters applied) divided by the number of covered base pairs of all samples shown as boxplots and grouped by accessions and pedigrees. Gd_UK_2004 and Gd_JP_St.Louis_SE have the lowest number of SNPs per covered base pair, Vx and Wi have the most.

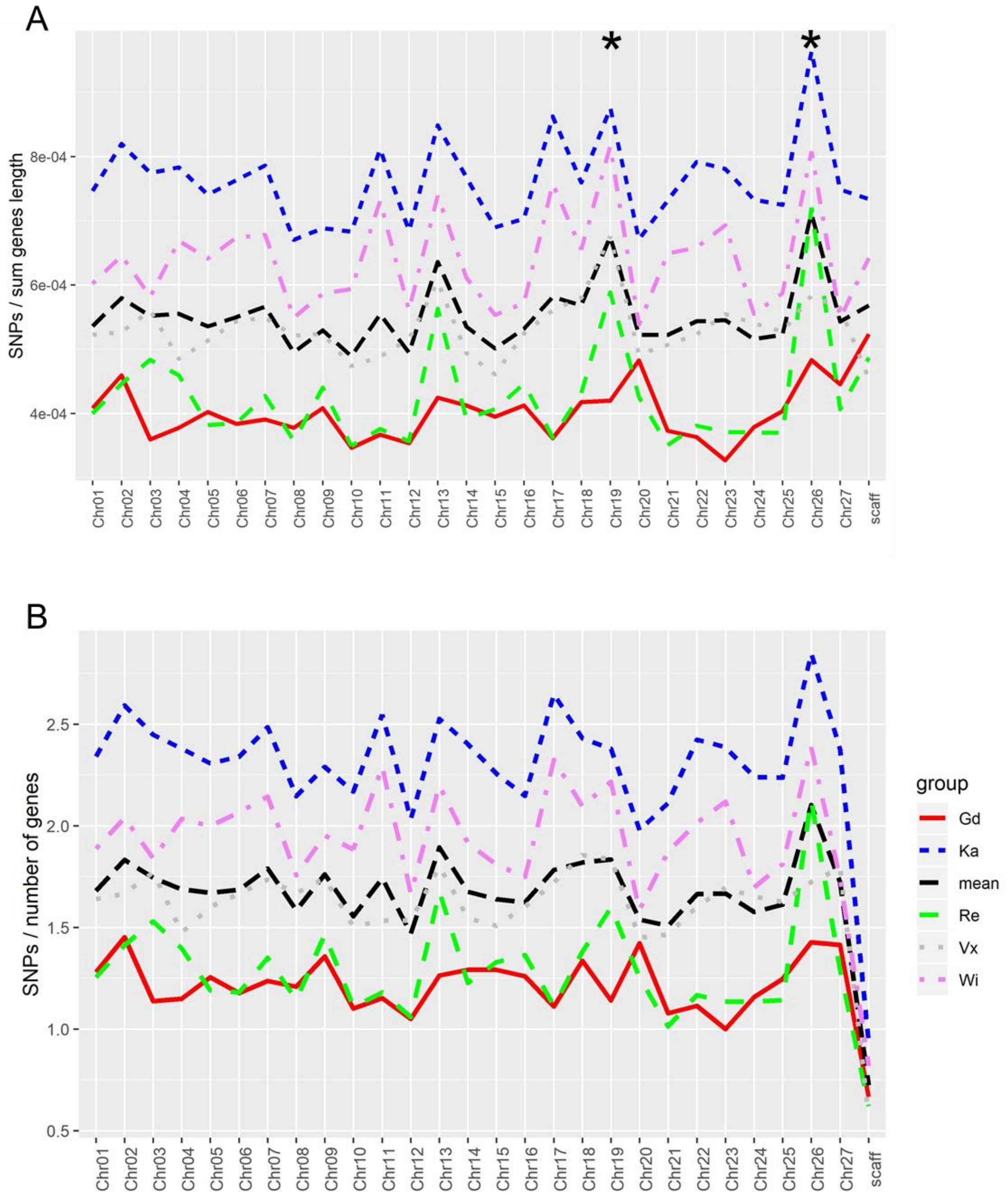

Figure S6: SNP to gene correlation.

The number of SNPs divided by the length of all genes per chromosome is shown in A), while in B) the SNPs are divided by the number of genes. Gd has an average of 1.2 SNPs per gene, while the SNP rate for Ka is almost double with 2.3 SNPs per gene. The black line represents the mean of all accessions. Chr19 and 26 appear to exhibit significantly more SNPs per gene length (Fisher's exact test, number of base pairs w/o SNPs for each individual chromosome, $p <= 0.05$).

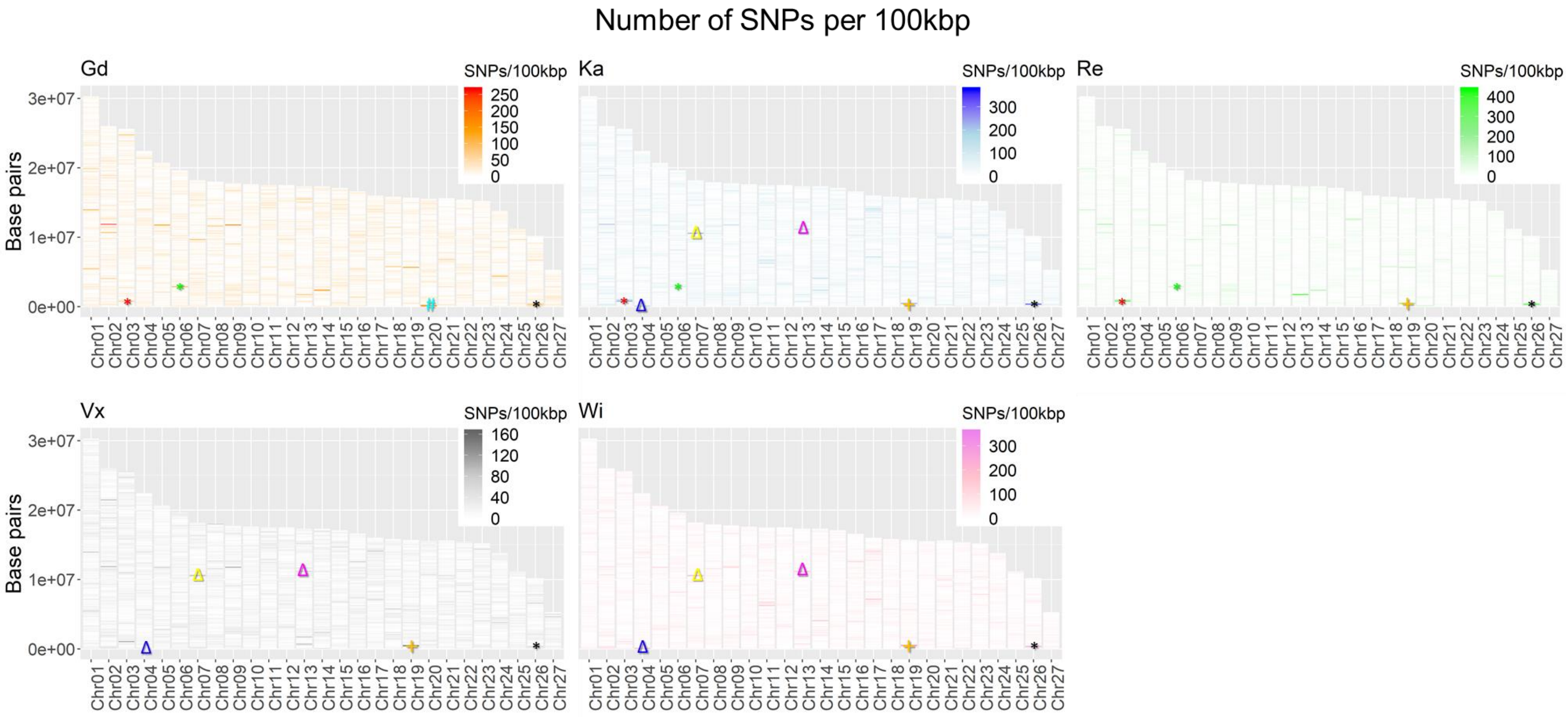

Figure S7: Number of SNPs per 100 kbp in each chromosome.

Each accession has its own shape of SNP hotspots. E.g. Gd has an exclusive 100kbp hotspot on Chr20 (starting at position 1) [light blue hash];. Some regions with high SNP numbers are shared between the accessions. Chr26 (start 300,000 bp) [black asterisk] is present in all accessions. Gd, Re and Ka share 100kbp hotspot regions on Chr03 (start 700,000 bp) [red asterisk] and one on Chr06 (start 2,800,000 bp) [green asterisk]; Ka, Wi, and Vx share regions on Chr04 (start 1 bp) [blue triangle], Chr07 (start 10,500,000 bp) [yellow triangle] and Chr13 (start 11,100,000 bp) [violet triangle]; all accessions except Gd share Chr19 (starting position 400,000 bp) [orange plus]. The full list is available in Supplementary File 2.

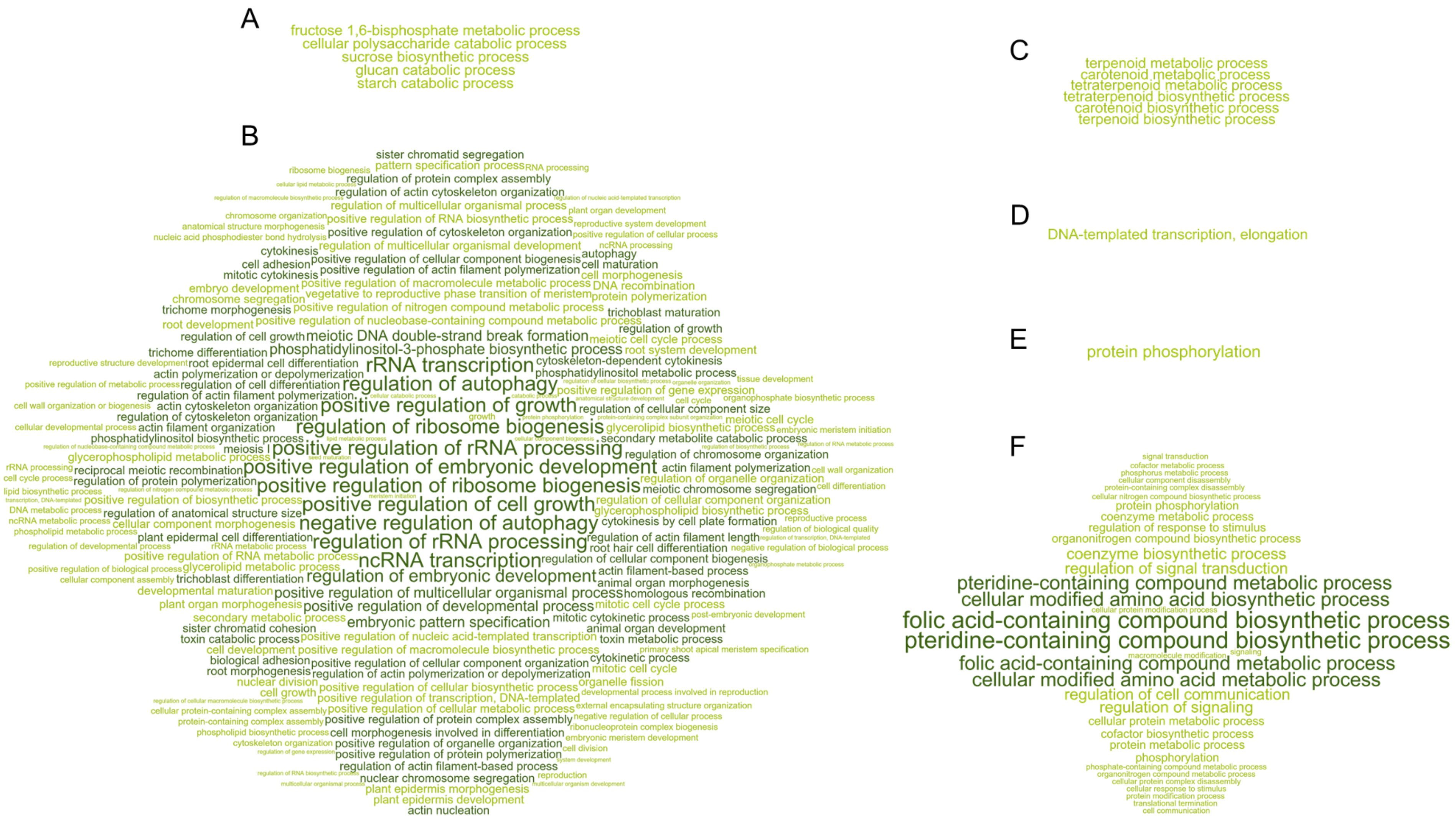

Figure S8: Word clouds of GO bias analyses of the 100 kbp hotspot regions marked in Fig. S7. A) Chr03 700 kbp, 12 genes B) Chr06 2,800 kbp, three genes C) Chr07 10,500 kbp, seven genes D) Chr13 11,100 kbp, 12 genes E) Chr19 400 kbp, 15 genes F) Chr26 300 kbp, 14 genes. The weight of the given terms was defined as the −log10(q-values) and the colour scheme used for the visualization was red for under-represented GO terms and green for those over-represented. Terms with stronger representation, i.e. weight >4, were represented with darker colours.

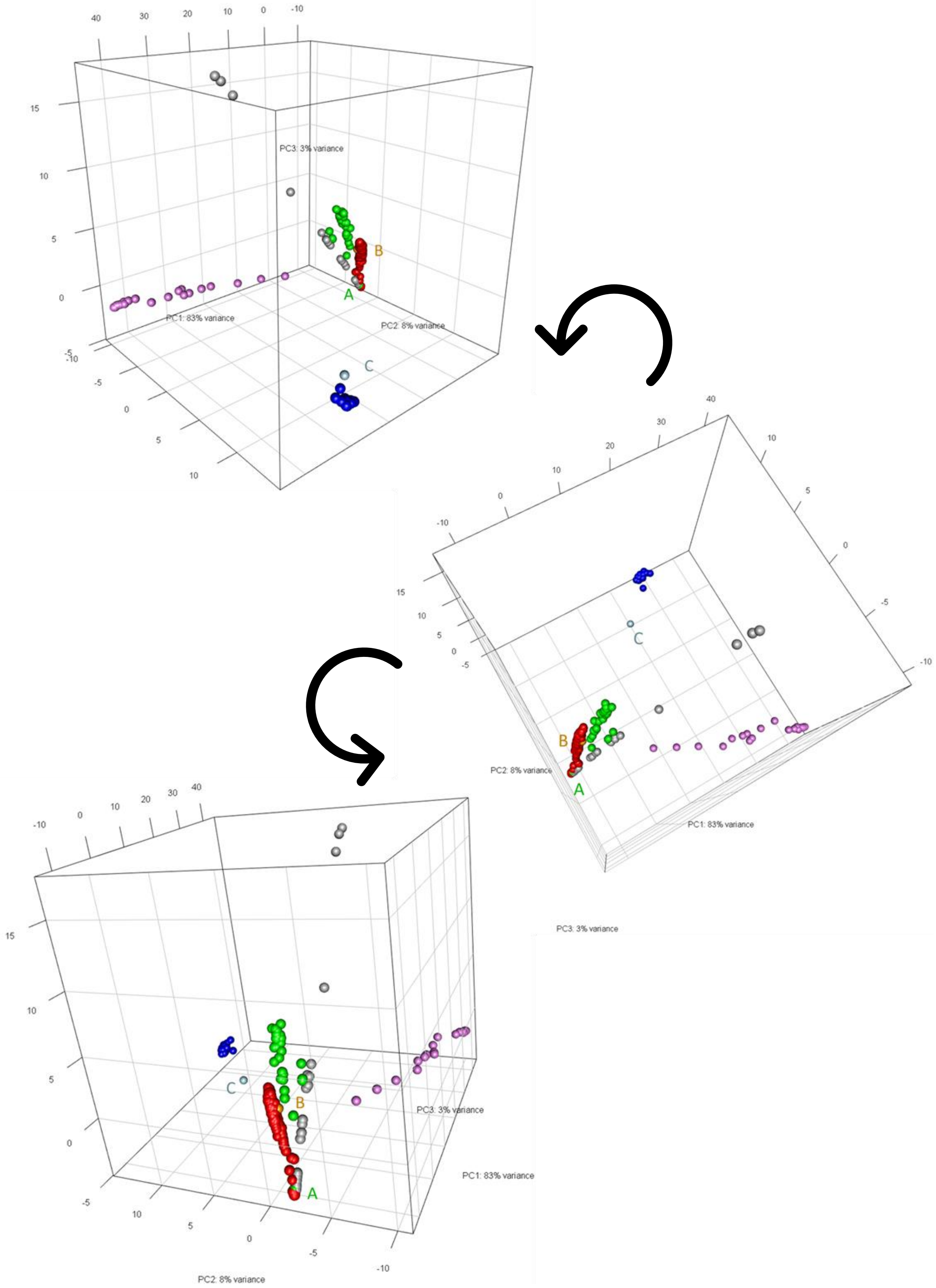

Figure S9: 3D PCA plot of filtered SNPs and InDels.

The PCA shows clustering by accessions similar to the SNP-only clustering (Fig. 4). The three plots are showing the cube rotated on its axis. Color scheme: Gd = red; Re = green; Ka = blue; Vx = grey; Wi = violet. The three outlier samples shown in Fig. 3 are labeled: A) CI_3, B) CIV_1 and C) XVIII_1.

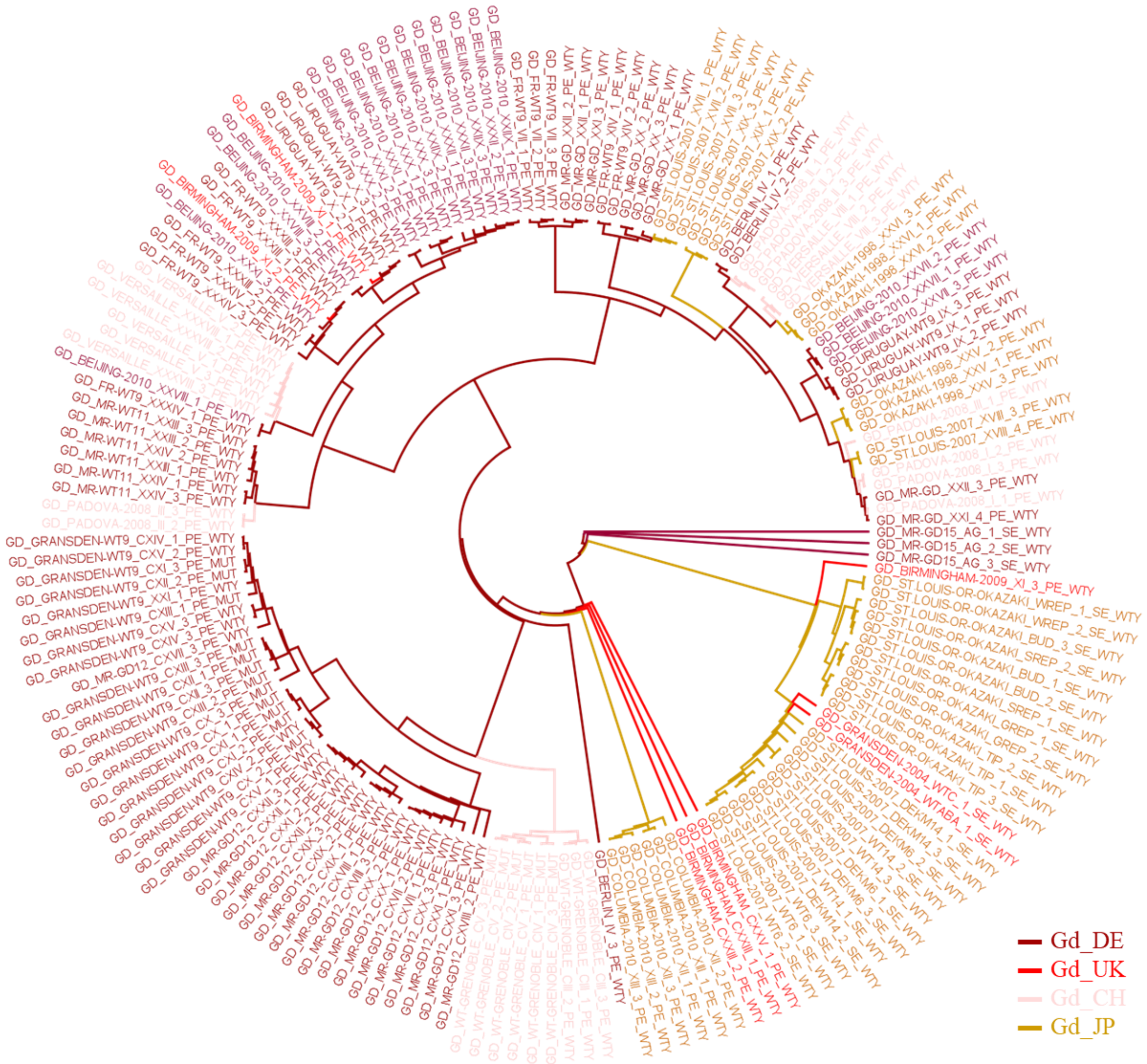

Figure S10: Gransden pedigree tree based on the artificial FASTA alignment.

Samples clustering by experiments can be observed. A clear clustering by pedigree is not observable.

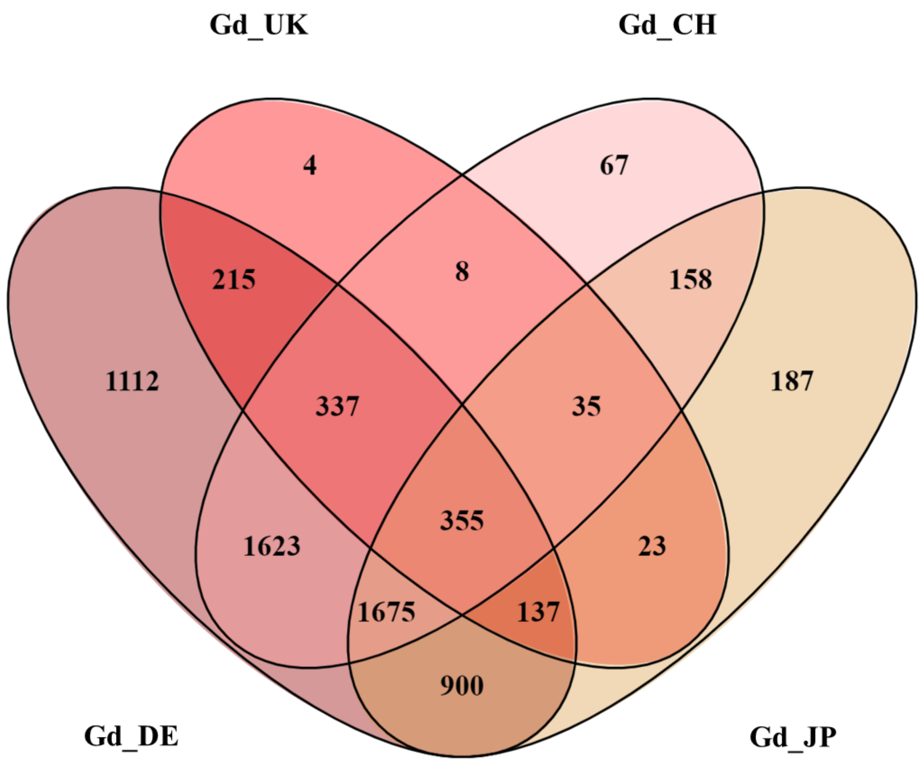

Figure S11: Intersection of all SNPs detected in the four Gransden pedigrees.

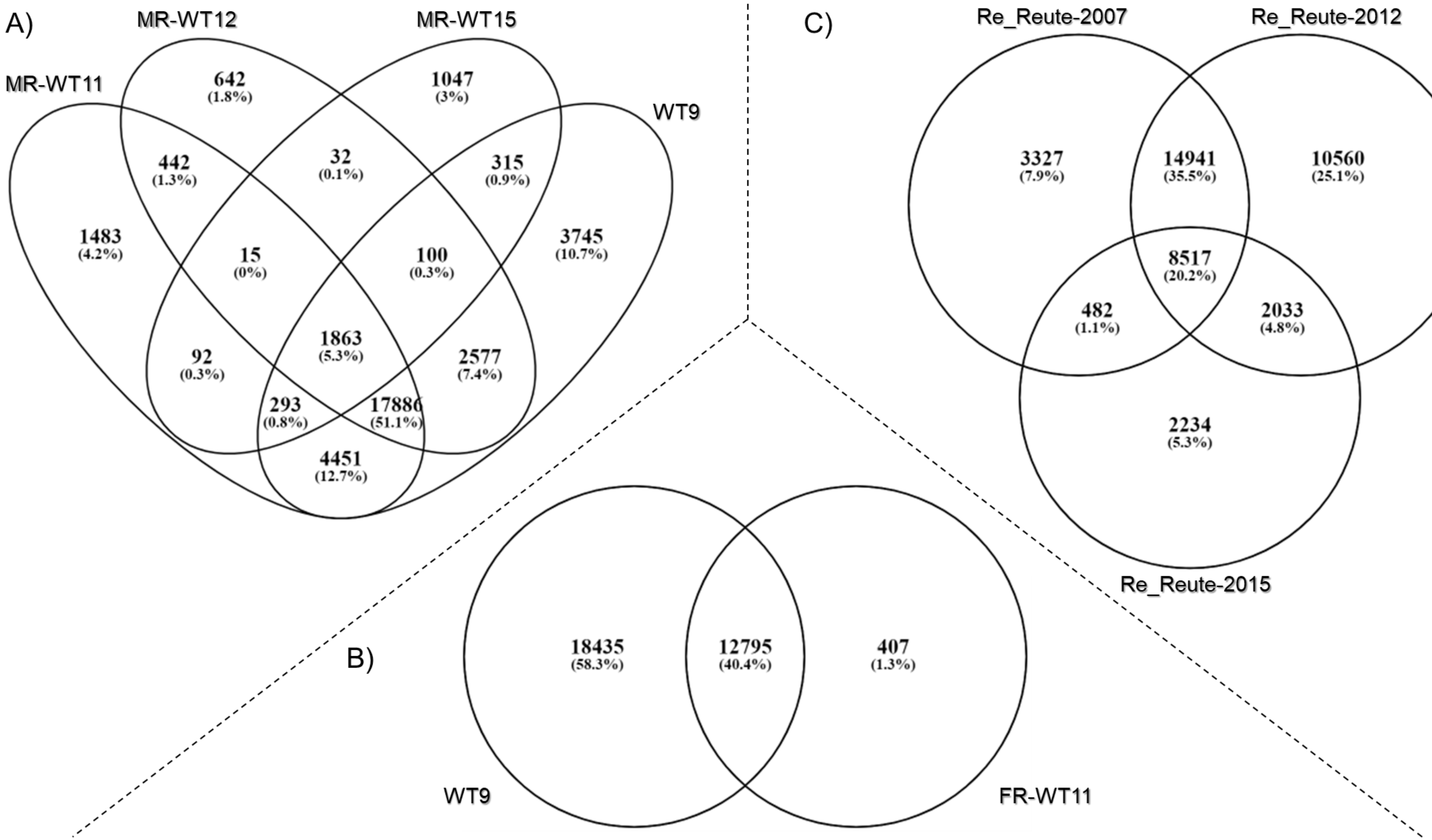

Figure S12: Intersection of all SNPs called for pedigrees with known propagation history. The SNPs were filtered by all three filter methods.

A) WT9 compared to Gd Marburg (MR) samples. B) WT9 compared to Freiburg (FR) sample WT-2011 and C) Three different pedigrees from accession Reute were compared.

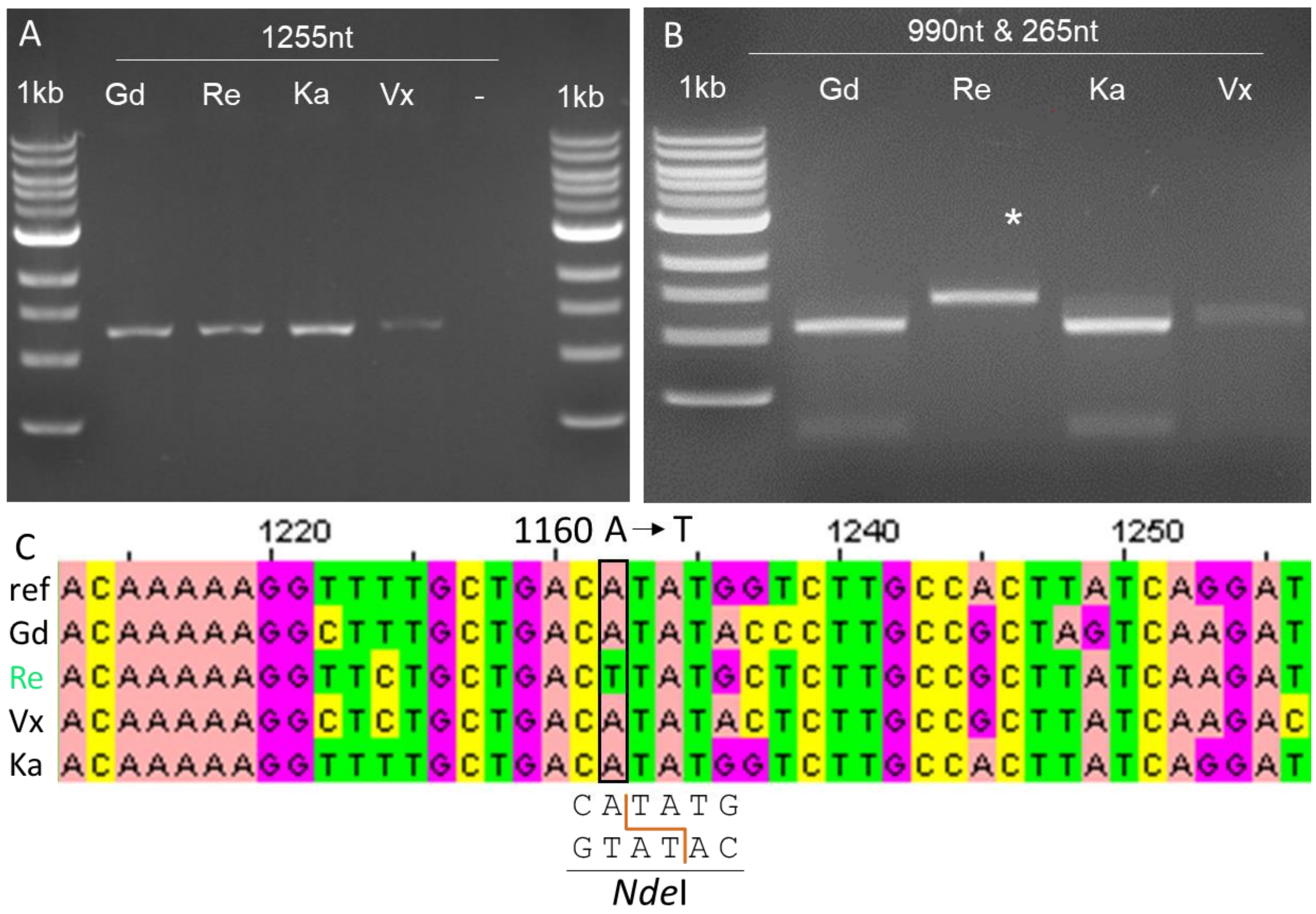

Figure S13: PCR and restriction analysis of the predicted SNP Re_c3_17747483_A-T. A: PCR product of 1,255nt was amplified from Gd_DE_MR, Re, Ka and Vx *P. patens* accessions. B: Restriction with *Nde*I resulted in two bands (990nt and 265nt) for Gd, Ka and Vx but not for Re (*). C: Alignment file of sequences derived from the PCR shows the presence of the SNP in Re, but not in the other three ecotypes affecting the indicated *Nde*I restriction site.

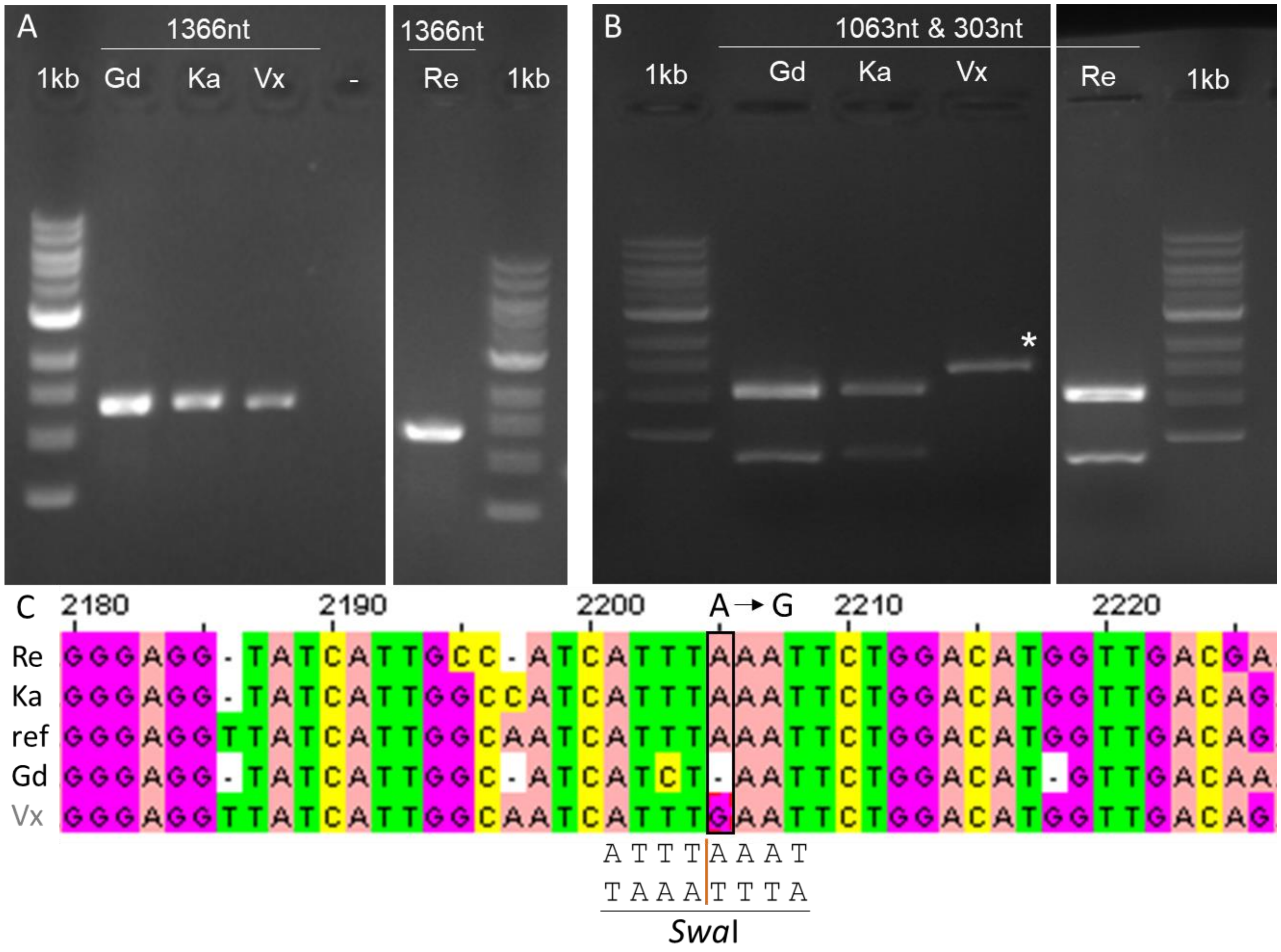

Figure S14: PCR and restriction analysis of the predicted SNP Vx_c3_2712099_A-G. A: PCR product of 1,366nt was amplified from Gd_DE_MR, Re, Ka and Vx *P. patens* accessions. B: Restriction with *Swa*I resulted in two bands for Gd, Ka and Re but not for Vx (*). C: Alignment file of sequences derived from the PCR shows the presence of the SNP in Vx, but not in the other three ecotypes.

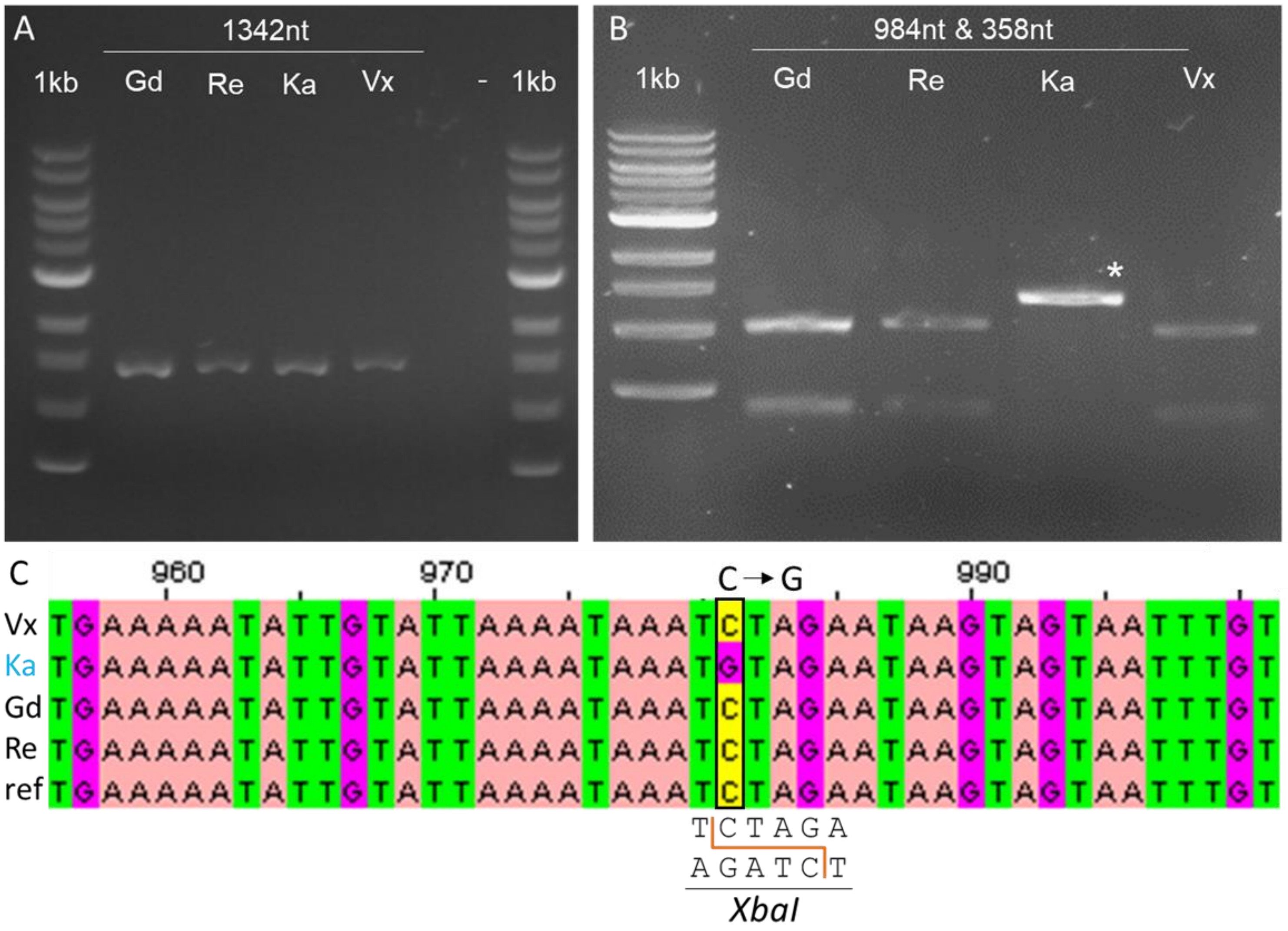

Figure S15: PCR and restriction analysis of the predicted SNP Ka_c1_25061888_C-G. A: PCR product of 1,342nt was amplified from Gd_DE_MR, Re, Ka and Vx *P. patens* accessions. B: Restriction with *Xba*I resulted in two bands for Gd, Vx and Re but not for Ka (*). C: Alignment file of sequences derived from the PCR shows the presence of the SNP in Ka, but not in the other three ecotypes.

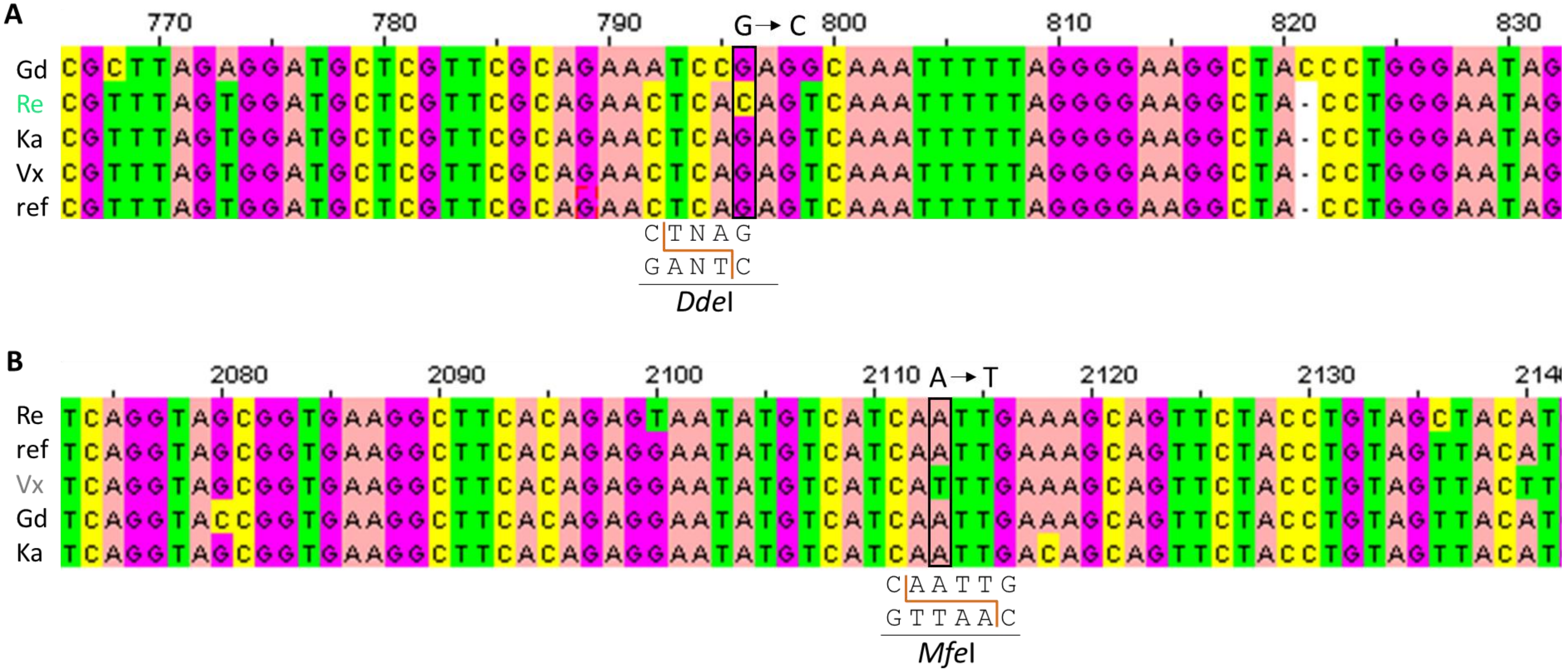

Figure S16: PCR and sequence analysis of the predicted SNPs for Re_c4_21933417_G-C and Vx_c13_4764050_A-T in four different accessions. A: Re_c4_21933417_G-C amplicons from Re, Vx, Gd and Ka aligned with the reference sequence (ref) show the presence of the predicted SNP, which affects the *Dde*I restriction enzyme site in Re (green). B: Vx_c13_4764050_A-T amplicons from Re, Vx, Gd and Ka aligned with the reference sequence (ref) show the presence of the predicted SNP, which affects the *Mfe*I restriction enzyme site in Vx (grey).

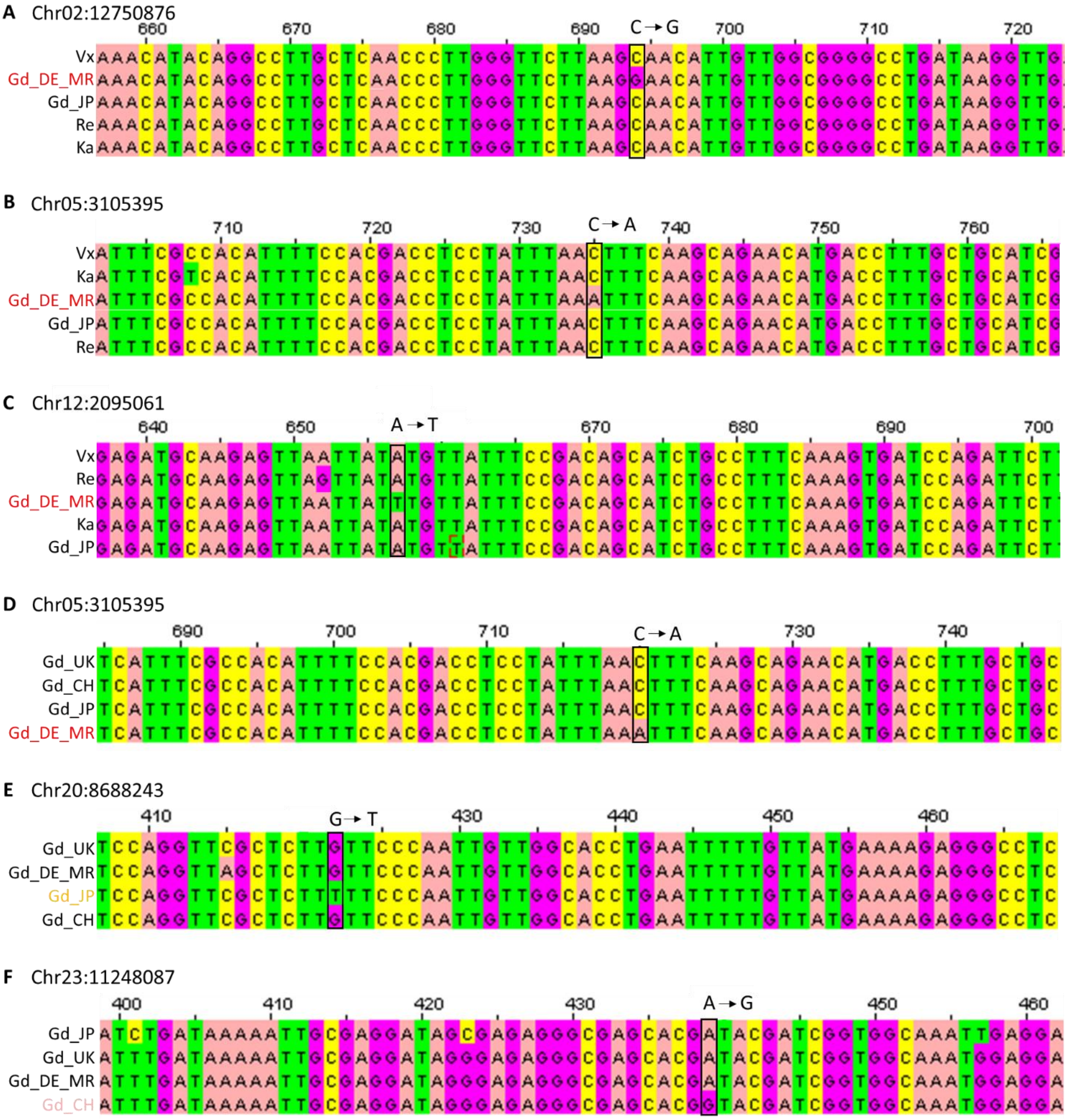

Figure S17: Sequence analysis of the predicted SNPs for Gd_DE_MR, Gd_JP and Gd_CH.

A: Sequencing results for the SNP Gd_DE_MR_c2_12750876_C-G amplicons derived from Gd_DE_MR, Re_DE, Vx and Ka were aligned. The SNP is present in the Gd_DE strain but not in the Gd_JP_Okazaki strain and the other P. patens accessions.

B: Sequencing results for the SNP Gd_DE _MR_c5_3105395_C-A amplicons derived from Gd_DE_MR, Re_DE, Vx and Ka were aligned. The SNP is present in the Gd_DE strain but not in the Gd_JP_Okazaki strain and the other P. patens accessions.

C: Sequences of Gd_DE_MR_c12_2095061_A-T amplicons from Gd_DE_MR, Re_DE, Vx and Ka were aligned. The SNP is present in the Gd_DE strain but not in the Gd_JP_Okazaki strain and the other P. patens accessions.

D: Sequences of Gd_DE_MR_c5_3105395_C-A amplicons from the Gd pedigrees Gd_UK, Gd_DE_MR, Gd_JP_St.Louis and Gd_CH were aligned. The SNP is present in the Gd_DE_MR strain but not in the other Gd strains.

E: Sequences of Gd_JP_20_8688243_G-T amplicons from the Gd pedigrees Gd_UK, Gd_DE_MR, Gd_JP_St.Louis and Gd_CH were aligned. The SNP is present in the Gd_JP strain but not in the other Gd strains.

F: Sequences of Gd_CH_c23_11248087_T-C amplicons from the Gd pedigrees Gd_UK, Gd_DE_MR, Gd_JP_St.Louis and Gd_CH were aligned. The SNP is present in the Gd_DE_MR strain but not in the other Gd strains.

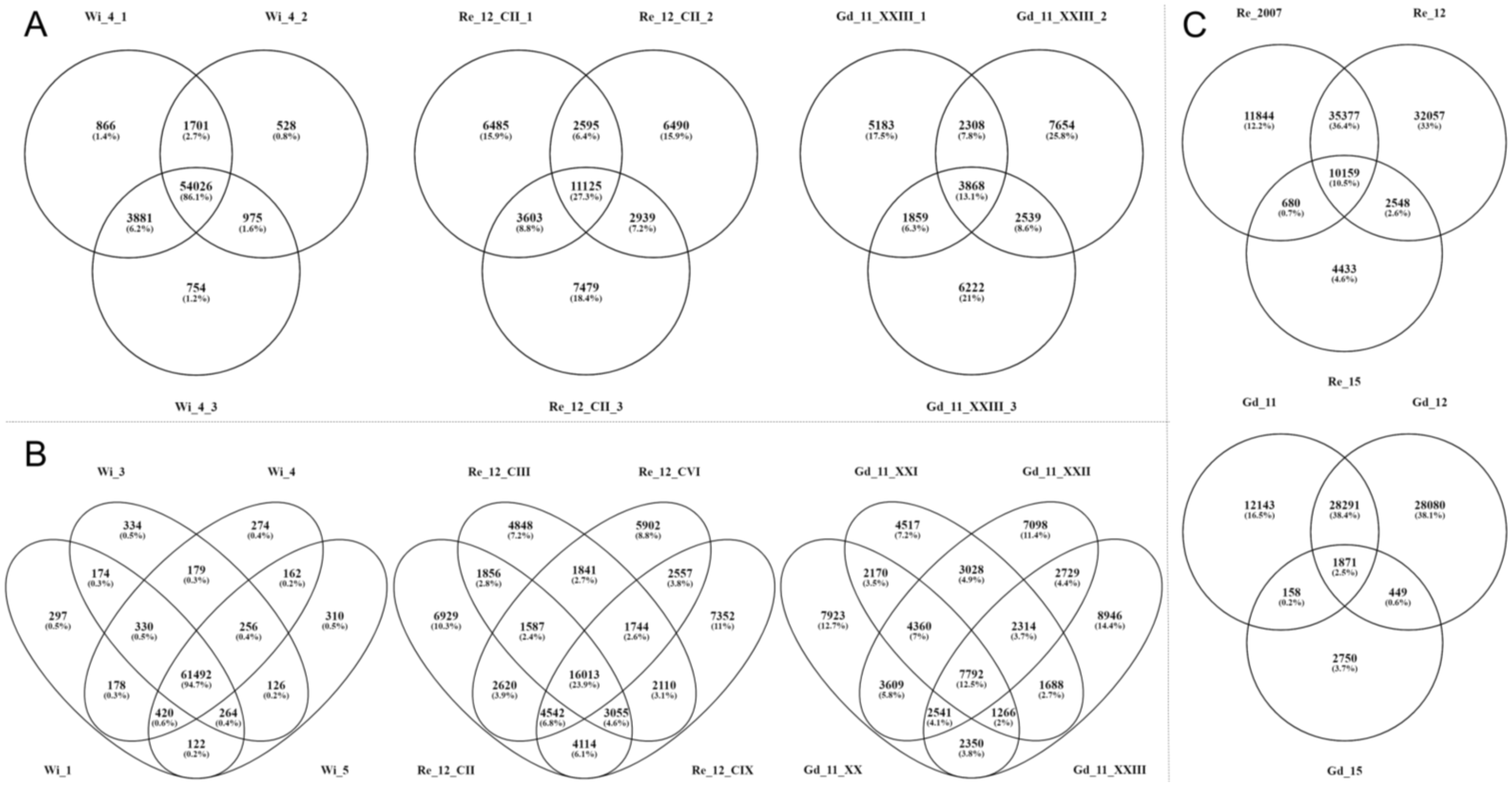

Figure S18: SNP intersection of Wi gDNA samples and selected Gd and Re pedigrees.

SNP intersection visualized by Venn diagrams. A) SNP intersection at sample replicate level. The leftmost diagram shows the SNP intersection of three sample replicates of the fourth Wi spore capsule. The middle and the right venn diagram show sample replicates of the experiment Re_REUTE-2012_CII and Gd_MR-WT11_XXIII. B) SNP intersection at experiment level. The left Venn diagram shows Wi gDNA SNPs of four spore capsule each consisting of five merged spore samples. The middle diagram shows the SNP intersection of all SNPs called in four different Reute_2012 experiments (CII, CIII, CVI and CIX). The rightmost Venn diagram shows the SNP intersection of four different Gd_DE_2011 experiments from Marburg (XX, XXI, XXII and XXIII). C) SNP intersection at pedigree level. The upper Venn diagram shows the SNP intersection of three Re generations (2007, 2012 and 2015). The lower Venn diagram shows the SNP intersection of three Gd_DE generations from Marbug (2011, 2012 and 2015).

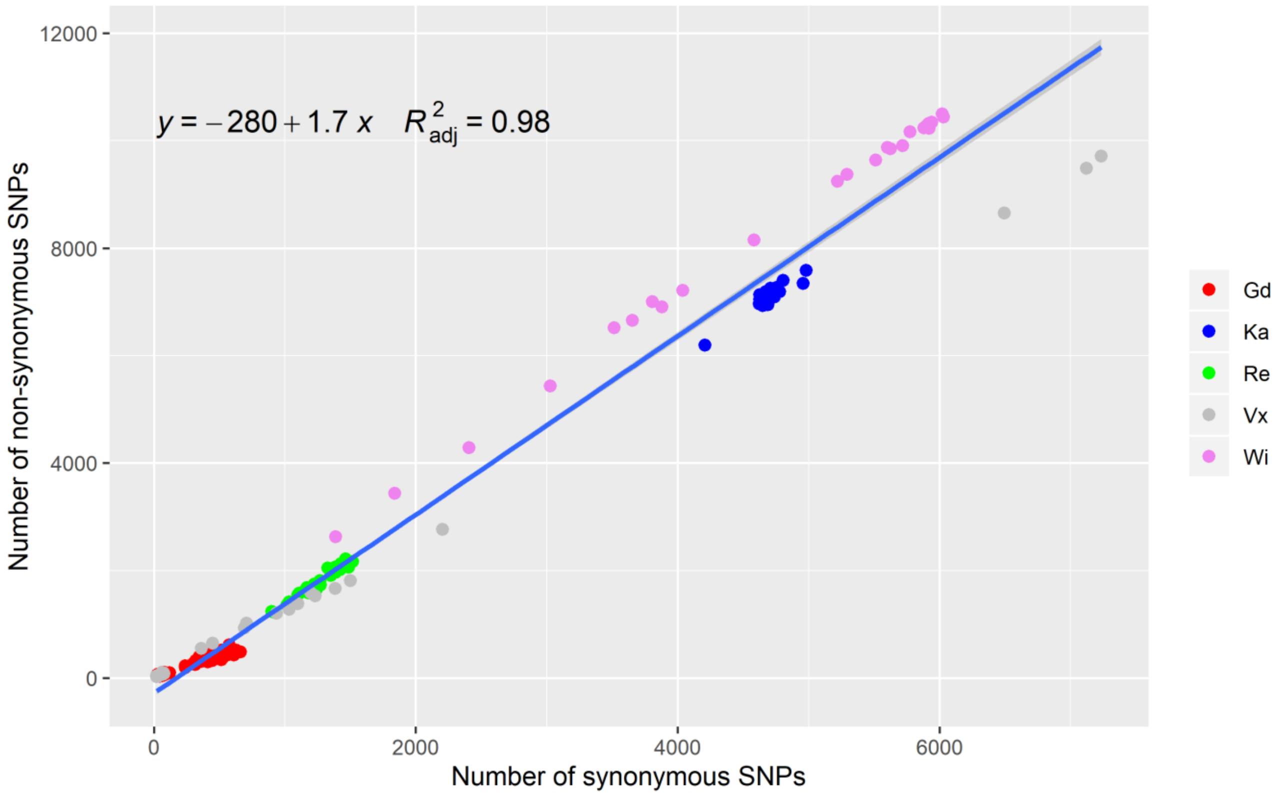

Figure S19: Ka / Ks plot of all samples.

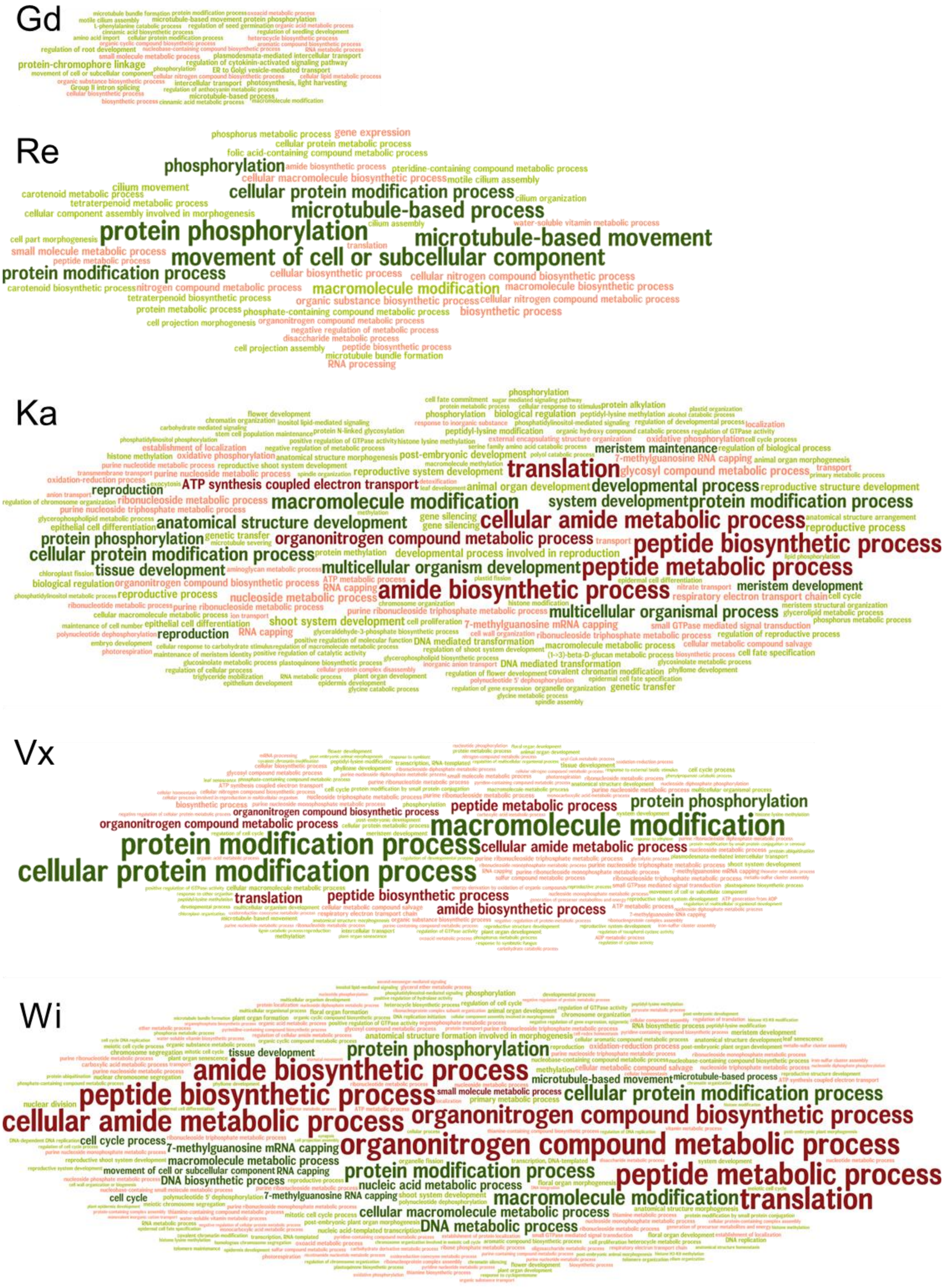

Figure S20: GO bias analysis of genes affected by non-synonymous SNPs. The word clouds are separated by accessions. The weight of the given terms was defined as the −log10(q-values) and the

colour scheme used for the visualization was red for under-represented GO terms and green for those over-represented. Terms with stronger representation, i.e. weight >4, were represented with darker colours.

Supplementary data, scripts

Script “rename_and_extraction”

```
#!/bin/bash

echo "Rename samples and extract Wi v3.3 SNPs"

# rename all vcf files
for IN in *.vcf; do mv $IN `awk -v name=$IN -v prefix=$prefix 'name~$1
{print $2"_"prefix".vcf"}' sample_translation.list`; done

# extract SNPs at gene regions based on gDNA samples
for IN in Wi_*.vcf; do bedtools intersect -u -a $IN -b Pp_v3.3.gff -header
> $IN".Pp_v3.3.vcf"; done

exit
```

Script “SNP_clustering”

```
#!/bin/bash

echo "SNP clustering"

prefix=$1  # surfix/run name
FI=$2      # samples supporting the SNP
DP=$3      # read depth
ADa=$4     # reads supporting the SNP

# fold change of the reads supporting the SNP
ADr=0.$(( ($ADa * 100) / $DP ))
AD=$ADa"x"$ADr


# clustering SNPs and InDels, 0/1/2-matrix
awk '{if(NR==FNR){for(i=5;i<=NF;i++){foo[$i]}; header[$1"_"$2"_"$3"_"$4]}
else {for(a in
foo){if($0~a){if((length($3)+length($4))>2){foo[a]=foo[a]"\t"2} else
{foo[a]=foo[a]"\t"1}} \
  else{foo[a]=foo[a]"\t"0}}} } END {printf "sample\t\t"; for(h in
header){printf h"\t"};printf "\n"; for(b in foo){split(b,c,"/"); print
c[1]"\t"foo[b]}}' \
  $prefix".plus_filter.filter"$FI".wo_indel.rna_snps"
$prefix".plus_filter.filter"$FI".wo_indel.rna_snps" | \
  sed 's/\t\n/\n/g' | sed 's/\t$//' >
$prefix".plus_filter.filter"$FI".wo_indel.0_1_2.rna_list"

# create SNP FASTA alignment file
awk '{if(NR==FNR){for(i=5;i<=NF;i++){foo[$i]}} else {for(a in
foo){if($0~a){foo[a]=foo[a]$4} else{foo[a]=foo[a]$3}}} } END {for(b in
foo){split(b,c,"/"); print ">"c[1]"\n"foo[b]}}' \
  $name".plus_filter.filter"$FI".wo_indel.rna_snps" \
  $name".plus_filter.filter"$FI".wo_indel.rna_snps" \
  > $name".plus_filter.filter"$FI".wo_indel.rna_snps.fasta"

exit
```

Script “SNP_filtering”

```
#!/bin/bash

echo "SNP filtering"

FI=$1       # samples supporting the SNP
DP=$2       # read depth
ADa=$3      # reads supporting the SNP
name=$4     # prefix/run name

# fold change of the reads supporting the SNP
ADr=0.$(( ($ADa * 100) / $DP ))
AD=$ADa"x"$ADr

# Merging all SNPs into one file.
# column 1-4: chromosome, position, reference, SNP
# column 5-n: vcf files containing the SNP
# Is a SNP passing the read coverage filter,
#    a '+' is added to the sample name
awk -v dp=$DP -v adr=$ADr -v ada=$ADa '$1!~"#" {split($10,a,":");
split(a[2],b,","); if(a[3]>=dp&&b[2]>=ada) {fc=b[2]/a[3];
if(fc>=adr){get_name=FILENAME"+"} else {get_name=FILENAME}} else
{get_name=FILENAME};
foo[$1"\t"$2"\t"$4"\t"$5]=get_name"\t"foo[$1"\t"$2"\t"$4"\t"$5]} END
{for(u in foo){print u"\t"foo[u]}}' Ka*vcf Gd*vcf Vx*vcf Re*vcf Wi*8.vcf >
$name".rna_snps"

# read coverage filter for all vcf files
for IN in *.vcf; do awk -v dp=$DP -v adr=$ADr -v ada=$ADa '$1!~"#"
{split($10,a,":"); split(a[2],b,","); if(a[3]>=dp&&b[2]>=ada)
{fc=b[2]/a[3]; if(fc>=adr) {print }}}' $IN > $IN".filterDP"$DP"AD"$AD ;
done

# support by at least $FI samples
awk -v FI=$FI 'NF>=4+FI {print }' $name".rna_snps" >
$name".filter"$FI".rna_snps"

# remove InDels
awk 'length($3)==1&&length($4)==1 {print }' $name".rna_snps" >
$name".wo_indel.rna_snps"

# extract all SNPs for each accession
for IN in Gd_ Re_ Ka_ Vx_ Wi_; do grep $IN $name".rna_snps" >
$IN$name".rna_snps"; done

# extract all SNPs for each Gd pedigree
grep -E "MR|Beij|FR|WT9|Berlin" Gd_$name".rna_snps" | grep "\+" >
Gd_DE.$name".plus_filter.rna_snps"
grep -E "Birm|2004_" Gd_$name".rna_snps" | grep "\+" >
Gd_UK.$name".plus_filter.rna_snps"
grep -E  "Padov|Versai|Greno" Gd_$name".rna_snps" | grep "\+" >
Gd_FR.$name".plus_filter.rna_snps"
grep -E "Colum|Okaza|Louis" Gd_$name".rna_snps" | grep "\+" >
Gd_JP.$name".plus_filter.rna_snps"

# extract all exclusive SNPs (here for Gd)
```

```
grep -Ev "Re_|Vx_|Wi_|As_|Ka_" $name".rna_snps" | awk '{print NF-4"\t"$0}'
| sort -k1V > Gd.exclusive

# extracting Gd pedigree exclusive SNPs (Gd.exclusive)
grep -Ev "MR|Bei|FR|WT9|Berlin|Birm|2004_|Padov|Versai|Greno" Gd.exclusive
| sort -V > Gd_JP.exclusive
grep -Ev "Padov|Versai|Greno|Colum|sdenE|Okaza|Louis|MR|Bei|FR|WT9|Berlin"
Gd.exclusive | sort -V > Gd_UK.exclusive
grep -Ev "MR|Bei|FR|WT9|Berlin|Birm|2004_|Colum|sdenE|Okaza|Louis"
Gd.exclusive | sort -V > Gd_FR.exclusive
grep -Ev "Birm|2004_|Padov|Versai|Greno|Colum|sdenE|Okaza|Louis"
Gd.exclusive | sort -V > Gd_DE.exclusive


exit
```

Supplementary References

Demko V, Perroud P-F, Johansen W, Delwiche CF, Cooper ED, Remme P, Ako AE, Kugler KG, Mayer KFX, Quatrano R, et al. 2014. Genetic Analysis of DEFECTIVE KERNEL1 Loop Function in Three-Dimensional Body Patterning in Physcomitrella patens. Plant Physiology.166:903-919.

Fernandez-Pozo N, Haas FB, Meyberg R, Ullrich KK, Hiss M, Perroud P-F, Hanke S, Kratz V, Powell AF, Vesty EF, et al. 2019. PEATmoss (Physcomitrella Expression Atlas Tool): a unified gene expression atlas for the model plant Physcomitrella patens. The Plant Journal.102:165-177.

Frank MH, Scanlon MJ. 2015. Cell-specific transcriptomic analyses of three-dimensional shoot development in the moss Physcomitrella patens. The Plant Journal.83:743-751.

Meyberg R, Perroud P-F, Haas FB, Schneider L, Heimerl T, Renzaglia KS, Rensing SA. 2020. Characterization of evolutionarily conserved key players affecting eukaryotic flagellar motility and fertility using a moss model. New Phytologist.n/a.

Perroud P-F, Haas FB, Hiss M, Ullrich KK, Alboresi A, Amirebrahimi M, Barry K, Bassi R, Bonhomme S, Chen H, et al. 2018. The Physcomitrella patens gene atlas project: large scale RNA-seq based expression data. The Plant journal : for cell and molecular biology.95:168-182.

Stevenson SR, Kamisugi Y, Trinh CH, Schmutz J, Jenkins JW, Grimwood J, Muchero W, Tuskan GA, Rensing SA, Lang D, et al. 2016. Genetic Analysis of *Physcomitrella patens* Identifies *ABSCISIC ACID NON-RESPONSIVE*, a Regulator of ABA Responses Unique to Basal Land Plants and Required for Desiccation Tolerance. The Plant Cell.28:1310-1327.

Szövényi P, Ullrich KK, Rensing SA, Lang D, van Gessel N, Stenøien HK, Conti E, Reski R. 2017. Selfing in Haploid Plants and Efficacy of Selection: Codon Usage Bias in the Model Moss Physcomitrella patens. Genome Biology and Evolution.9:1528-1546.
